# Energetic and Economic Aspects of Rebound, Part II: Applications of the Framework

**DOI:** 10.1177/01956574251331966

**Published:** 2025-07-24

**Authors:** Matthew Kuperus Heun, Gregor Semieniuk, Paul E. Brockway

**Affiliations:** 1 Engineering Department, Calvin University, Grand Rapids, MI, USA; 2 Sustainability Research Institute, School of Earth and Environment, University of Leeds, Leeds, UK; 3 Faculty of Economic and Management Science, School for Public Leadership, Stellenbosch University, Stellenbosch, South Africa; 4 School of Public Policy & Department of Economics, University of Massachusetts Amherst, Amherst, MA, USA; 5 World Bank, Washington, DC, USA

**Keywords:** energy efficiency, energy rebound, energy services, microeconomic rebound, substitution and income effects, macroeconomic rebound

## Abstract

Widespread implementation of energy efficiency is a key greenhouse gas emissions mitigation measure, but rebound can “take back” energy savings. However, the absence of solid analytical foundations hinders empirical determination of rebound magnitudes. In Part I, we developed foundations of a rigorous, analytical, consumer-sided rebound framework that is approachable for both energy analysts and economists. In this paper (Part II), we develop energy, expenditure, and consumption planes, a novel, mutually consistent, and numerically precise way to visualize and illustrate rebound. Further, we operationalize the macro factor ( k ) for macroeconomic rebound. Using the framework and rebound planes, we calculate and show total rebound (using k=3 ) for two examples: energy efficiency upgrades of a car (56.2%) and an electric lamp (67.0%). We also calculate rebound when extending the framework to include an energy price effect. Finally, we provide information about new open-source software tools for calculating rebound magnitudes and visualizing rebound effects.

**JEL Classification:** O13, Q40, Q43

## 1. Introduction

In Part I of this two-part paper, we argued that improved clarity is needed about energy rebound. We said that

[a] description of rebound [is needed] that is (i) consistent across energy, expenditure, and consumption aspects, (ii) technically rigorous and (iii) approachable from both sides (economics and energy analysis). … In other words, the finance and human behavior aspects of rebound need to be presented in ways energy analysts can understand. And the energy aspects of rebound need to be presented in ways economists can understand.

**Figure 1.**
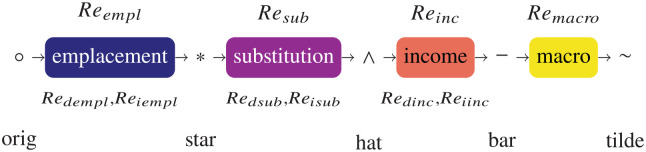
Flowchart of rebound effects and decorations.

To help improve clarity in the rebound field, we developed in Heun et al. (2025) foundations for a rigorous analytical framework, one that is tractable for both energy analysts and economists. Three aspects of rebound are analyzed in the framework: energy, expenditure, and consumption. The framework contains both direct and indirect rebound and four rebound effects (emplacement, substitution, income, and macro) between five stages (°, *, ∧, −, and ~). Rebound terms and symbol decorations are shown in Figure 1. (See Table 1 in Part I for details. See Appendix A for nomenclature.)

**Table 1.** Car Example: Vehicle Parameters.

Description parameters [units]	Ford Fusion (gasoline)	Ford Fusion (hybrid EV)	Data sources and notes
Fuel economy $\eta^{{}^{\circ}}$ , $\eta^{*}$ [mpg]	25	42	Combined cycle mpg value taken from Thecarconnection.com (2020), for Titanium FWD 2020 model with Intercooled I-4, 2.0 L engine. Combined cycle mpg value taken from Thecarconnection.com (2020), for Titanium FWD 2020 model with Gas/Electric I-4, 2.0 L engine.
Undiscounted capital expenditure rate ${\overset{\cdot }{C}}_{\mathrm{cap}}^{{}^{\circ}}$ , ${\overset{\cdot }{C}}_{\mathrm{cap}}^{*}$ [$/yr]	2,533	2,518	Fourteen year annual, averaged capital costs = purchase cost/ $t_{\mathrm{life}}$ . Ford Fusion gasoline costs from Edmunds.com (2020a). Ford Fusion Hybrid car costs from Edmunds.com (2020b).
Lifespan $t_{\mathrm{life}}^{{}^{\circ}}$ , $t_{\mathrm{life}}^{*}$ [yr]	14	14	Lifetime taken as fourteen years, based on thirteen to seventeen years for U.S. cars from Berla.com (2016) and fourteen years for UK cars from Society of Motor Manufacturers and Traders (2020).
Embodied energy $E_{\mathrm{emb}}^{{}^{\circ}}$ , $E_{\mathrm{emb}}^{*}$ [MJ]	34,000	40,000	34,000 MJ for conventional Ford Fusion gasoline car taken from Argonne National Laboratory, Energy Systems Division (2010). We assume an additional 6,000 MJ added for Ford Fusion Hybrid Electric Vehicle (HEV) battery, as HEV typically adds 10% to 25% to total LCA energy of vehicle manufacture (Onat, Kucukvar and Tatari 2015). Battery lifetime assumed same as car lifetime, based on Nordelöf et al. (2014) and Onat, Kucukvar and Tatari (2015).
Operations and maintenance expenditure rate ${\overset{\cdot }{C}}_{\mathrm{OM}}^{{}^{\circ}}$ , ${\overset{\cdot }{C}}_{\mathrm{OM}}^{*}$ [$/yr]	5,050	4,779	Lifetime (fourteen year) annual, averaged operation and maintenance (O&M) costs = sum of insurance, maintenance, repairs, taxes, and fees (excluding financing, depreciation, fuel). Five-year Ford Fusion O&M costs from Edmunds.com (2020a). Five-year Ford Fusion Hybrid O&M costs from Edmunds.com (2020b). Extrapolation of O&M costs for years 6 to 14 based on Djokic et al. (2015).
Disposal cost $C_{d}^{{}^{\circ}}$ , $C_{d}^{*}$ [$]	−300	−300	Salvage value (negative cost) taken from Junk Car Medics (2024)
Ops., maint., and disposal expenditure rate, discounted ${\overset{\cdot }{C}}_{\mathrm{OMd}}^{{}^{\circ}}$ , ${\overset{\cdot }{C}}_{\mathrm{OMd}}^{*}$ [$/yr]	5,033	4,762	Sum of annualized operations, maintenance, and disposal costs.

In this paper (Part II), we make further progress toward the goal of clarity with five contributions. First, we develop a new way to communicate components and mechanisms of rebound via mutually consistent and numerically precise visualizations of rebound effects in energy, expenditure, and consumption planes. Second, we calculate the macro rebound effect via a macro factor ( k ) selected to be 3. Third, we apply the framework to two energy efficiency upgrades (EEUs; a car and an electric lamp) with detailed explication of numerical results for the examples. Fourth, we apply the framework to calculate numerical values for an energy price effect. Finally, we provide information about new open source software tools for calculating and visualizing rebound for any energy efficiency upgrade.

The remainder of this paper is structured as follows. Section 2 describes data for the examples, our method of visualizing rebound, and open source software tools for calculating and visualizing rebound. Section 3 provides results for two examples: energy efficiency upgrades to a car and an electric lamp. Section 4 operationalizes the macro factor ( k ) and discusses results, and Section 5 concludes.

## 2. Data and Methods

This section contains data for the examples (Section 2.1), an explication of our new method for visualizing rebound effects and magnitudes (Section 2.2), and a description of new open source software tools for rebound calculations and visualization (Section 2.3).

### 2.1. Data

To demonstrate application of the rebound analysis framework developed in Part I, we analyze two examples: energy efficiency upgrades to a car and an electric lamp. The examples are presented with much detail to support our goal of helping to advance clarity for the process of calculating the magnitude of rebound effects. Here, we collect parameter values for the equations to calculate nine rebound components: $Re_{\mathrm{dempl}}$ , $Re_{\mathrm{emb}}$ , $Re_{\mathrm{OM}}$ , $Re_{d}$ , $Re_{\mathrm{dsub}}$ , $Re_{\mathrm{isub}}$ , $Re_{\mathrm{dinc}}$ , $Re_{\mathrm{iinc}}$ , and $Re_{\mathrm{macro}}$ . Total rebound ( $Re_{\mathrm{tot}}$ ) is given by the sum of the above components or equivalently by equation (35) of Part I.

#### 2.1.1. Data for Car Example

For the first example, we consider the purchase of a more fuel efficient car, namely a gasoline-electric Ford Fusion Hybrid car, to replace a conventional gasoline Ford Fusion car. The cars are matched as closely as possible, except for the inclusion of an electric battery in the hybrid car. The car case study features a larger initial capital investment ( $C_{\mathrm{cap}}^{{}^{\circ}}<C_{\mathrm{cap}}^{*}$ ) for the long-term benefit of decreased energy service costs ( ${\overset{\cdot }{C}}_{s}^{{}^{\circ}}>{\overset{\cdot }{C}}_{s}^{*}$ ).

We require three sets of data. First, basic car parameters are summarized in Table 1. Second, we require several general economic parameters, mainly relating to the U.S. economy and personal finances of a representative U.S.-based user shown in Table 2. Third, we require elasticity parameters, as given in Table 3.

**Table 2.** Car Example: Economic Parameters (2020).

Description parameter [units]	Value	Data sources and notes
Distance driven prior to upgrade ${\overset{\cdot }{q}}_{s}^{{}^{\circ}}$ [miles/yr]	12,416	Average U.S. vehicle miles/yr, calculated from Carinsurance.com (2019). This is slightly lower than the average driver miles/yr (13,476; U.S. Department of Transportation 2018), as there are more registered U.S. vehicles than drivers.
Real median personal income U.S., in 2018 [$/yr]	34,317	Taken from Federal Reserve Bank of St Louis (2019).
U.S. 2018 disposable income/real income (minus current taxes) [–]	0.88319	Taken from U.S. Bureau of Economic Analysis (BEA) National and Products Accounts (NIPA) Table 2.1. Personal Income and Its Disposition (U.S. Bureau of Economic Analysis 2020).
Share of savings from 2018 disposable income [–]	0.07848	Taken from U.S. Bureau of Economic Analysis (BEA) National and Products Accounts (NIPA) Table 2.1. Personal Income and Its Disposition (U.S. Bureau of Economic Analysis 2020).
Personal consumption in 2018 $\overset{\cdot }{M}$ [$/yr]	27,930	Calculation: ($34,317/yr)(0.88319)(1−0.07848)
Price of gasoline $p_{E}$ [$/gallon]	2.63	Source: U.S. Energy Information Administration (2020b)
Fractional spend on original energy service $f_{\overset{\cdot }{C}s}^{{}^{\circ}}$ [–]	0.066	Calculation: $1,306 (spend on energy service)/[$19,115 (other goods) + $1,306 (energy service)] = 0.064, where spend on energy service = 12,416 miles/25 mpg x $2.63/gallon = $1,306.
Real discount rate r [1/yr]	0.03	Taken from Federal Reserve St. Louis for seventy-two month car loan rate, which averaged 5% before the 2022 interest rate raises. Subtracting 2% inflation gives 3% real interest rate, by which we discount. (Board of Governors of the Federal Reserve System (U.S.) 2024)
Macro factor k [–]	1.0	An initial value. See Section 4.1 for additional details.

**Table 3.** Car Example: Elasticity Parameters.

Description parameter [units]	Value	Data sources and notes
Uncompensated own price elasticity of car use demand $\varepsilon_{{\overset{\cdot }{q}}_{s},p_{s}}^{{}^{\circ}}$ [–]	-0.2	We adopt -0.2 as our baseline value, based on U.S. studies including Gillingham (2020) who estimated a value of -0.1, Goetzke and Vance (2018) who estimated values between -0.05 and -0.23, and Parry and Small (2005) who estimated values between -0.1 and -0.3. For comparison, Borenstein (2015) uses values of -0.1 to -0.4 based on Parry and Small (2005).
Compensated price elasticity of car use demand $\varepsilon_{{\overset{\cdot }{q}}_{s},p_{s},c}^{{}^{\circ}}$ [–]	-0.134	Calculated via the Slutsky Equation (equation (172) in Part I).
Compensated cross price elasticity of demand for other goods $\varepsilon_{{\overset{\cdot }{q}}_{g},p_{s},c}^{{}^{\circ}}$ [–]	0.009	Calculated via equation (178) in Part I.
Income elasticity of demand for car use $\varepsilon_{{\overset{\cdot }{q}}_{s},\overset{\cdot }{M}}^{{}^{\circ}}$ [–]	1.0	Follows from CES utility function.
Income elasticity of demand for other goods $\varepsilon_{{\overset{\cdot }{q}}_{g},\overset{\cdot }{M}}^{{}^{\circ}}$ [–]	1.0	Follows from CES utility function.

#### 2.1.2. Data for Lamp Example

For the second example, we consider purchasing a Light Emitting Diode (LED) electric lamp to replace a baseline incandescent electric lamp. Both lamps are matched as closely as possible in terms of energy service delivery (measured in lumen output per lamp), the key difference being the energy required to provide that service. The LED lamp has a low initial capital investment rate when spread out over the lifetime of the lamp (less than the incumbent incandescent lamp) and a long-term benefit of decreased direct energy expenditures at approximately the same energy service delivery rate (lm hr/yr).

Again, three sets of data are required. First, basic lamp parameters are summarized in Table 4. Second, several general economic parameters, mainly relating to the U.S. economy and personal finances of a representative U.S.-based user are given in Table 5. Third, we require the elasticity parameters, as shown in Table 6.

**Table 4.** Lamp Example: Electric Lamp Parameters.

Description parameters [units]	Incandescent lamp	LED lamp	Data sources and notes
Lamp efficiency $\eta^{{}^{\circ}}$ , $\eta^{*}$ [lmċhr/Wċhr]	8.83	81.8	Incandescent: 530 lm output/60 W energy input. LED: 450 lm output/5.5 W energy input.
Undiscounted capital expenditure rate $C_{\mathrm{cap}}^{{}^{\circ}}$ , ${\overset{\cdot }{C}}_{\mathrm{cap}}^{*}$ [$/yr]	1.044	0.121	Purchase costs: $1.88 for incandescent lamp from HomeDepot.com (2020b), and $1.21 for LED lamp from HomeDepot.com (2020a).
Lifespan $t_{\mathrm{life}}^{{}^{\circ}}$ , $t_{\mathrm{life}}^{*}$ [yr]	1.8	10	Based on assumed three hour/day from HomeDepot.com (2020b) and HomeDepot.com (2020a).
Life cycle analysis (LCA) embodied energy $E_{\mathrm{emb}}^{{}^{\circ}}$ , $E_{\mathrm{emb}}^{*}$ [MJ]	2.20	6.50	Base document: Table 4.5 Manufacturing Phase Primary Energy (MJ/20 million lmċhr), contained in U.S. DoE Life-cycle assessment of energy and environmental impacts of LED lighting products (U.S. Department of Energy 2012). Incandescent lamp: LCA energy = 42.2 MJ/20 million lmċhr. Lifetime output = 530 lm x 3 hr/day x 365 days/yr x 1.8 yr = 1,044,630 lmċhr. Thus LCA energy/lamp = 42.2 x 1.0446/20 = 2.20 MJ. LED lamp: LCA energy = 132 MJ/20 Million lmċhr for pack of 5 LED lamps. Lifetime output = 450 lm x 3 hr/day x 365 days/yr x 10 yr = 4,926,405 lmċhr. Thus LCA energy/lamp = 132 MJ/5 x 4.9264/20 = 6.5 MJ.
Operations and maintenance expenditure rate ${\overset{\cdot }{C}}_{\mathrm{OM}}^{{}^{\circ}}$ , ${\overset{\cdot }{C}}_{\mathrm{OM}}^{*}$ [$/yr]	0	0	Lifetime annual, averaged operations and maintenance (O&M) costs. Once installed assumed 0. Note: O&M costs exclude fuel (i.e., electricity) costs.
Disposal cost $C_{d}^{{}^{\circ}}$ , $C_{d}^{*}$ [$]	0	0	Disposal cost assumed negligible (local/doorstep recycling facility).
Ops., maint., and disposal expenditure rate, discounted ${\overset{\cdot }{C}}_{\mathrm{OMd}}^{{}^{\circ}}$ , ${\overset{\cdot }{C}}_{\mathrm{OMd}}^{*}$ [$/yr]	0	0	Sum of annualized operations, maintenance, and disposal costs.

**Table 5.** Lamp Example: Economic Parameters (2020).

Description parameter [units]	Value	Data sources and notes
Lighting consumption prior to upgrade ${\overset{\cdot }{q}}_{s}^{{}^{\circ}}$ [lmċhr/yr]	580,350	Calculation: (530 lm) (3 hrs/day) (365 days/yr).
Real median personal income U.S. in 2018 [$/yr]	34,317	Refer to Table 2.
U.S. 2018 disposable income/real income (minus current taxes) [–]	0.88319	Refer to Table 2.
Share of savings from 2018 disposable income [–]	0.07848	Refer to Table 2.
Personal consumption in 2018 $\overset{\cdot }{M}$ [$/yr]	27,930	Calculation: ($34,317/yr)(0.88319)(1−0.07848) .
Price of electricity $p_{E}$ [$/kWċhr]	0.1287	U.S. 2018 average U.S. household electricity price (U.S. Energy Information Administration 2020a).
Fractional spend on original energy service $f_{\overset{\cdot }{C}s}^{{}^{\circ}}$ [–]	0.0003028	Calculation: $8.5/yr (spend on energy service)/[$27,920/yr (other goods) + $8.5/yr (energy service)] = 0.0003028, where spend on energy service = 580,350 lmċhr/yr/8.83 lm/W/1,000 W/kW x $0.1287/kWċhr = $8.5/yr. Note: this is the energy service from a single lamp.
Real discount rate r [1/yr]	0.03	Taken from Federal Reserve St. Louis for seventy-two month car loan rate, which averaged 5% before the 2022 interest rate raises. Subtracting 2% inflation gives 3% real interest rate, by which we discount. (Board of Governors of the Federal Reserve System (U.S.) 2024)
Macro factor k [–]	1.0	An initial value. See Section 4.1 for additional details.

**Table 6.** Lamp Example: Elasticity Parameters.

Description parameter [units]	Value	Data sources and notes
Uncompensated own price elasticity of lighting demand $\varepsilon_{{\overset{\cdot }{q}}_{s},p_{s}}^{{}^{\circ}}$ [–]	−0.4	We adopt −0.4 as our baseline value, as the average of last fifty years from Fouquet (2014, Figure 4). For comparison, Borenstein (2015) uses a range of −0.4 to −0.8, based on Fouquet and Pearson (2011).
Compensated own price elasticity of lighting demand $\varepsilon_{{\overset{\cdot }{q}}_{s},p_{s},c}^{{}^{\circ}}$ [–]	−0.3997	Calculated via the Slutsky Equation (equation (172) in Part I).
Compensated cross price elasticity of demand for other goods $\varepsilon_{{\overset{\cdot }{q}}_{g},p_{s},c}^{{}^{\circ}}$ [–]	0.00012	Calculated via equation (178) in Part I.
Income elasticity of lighting demand $\varepsilon_{{\overset{\cdot }{q}}_{s},\overset{\cdot }{M}}$ [–]	1.0	Follows from CES utility function.
Income elasticity of demand for other goods $\varepsilon_{{\overset{\cdot }{q}}_{g},\overset{\cdot }{M}}$ [–]	1.0	Follows from CES utility function.

### 2.2. Visualization

A rigorous rebound analysis should track energy, expenditure, and consumption aspects of rebound at the device (direct rebound) and elsewhere in the economy (indirect rebound) across adjustments for all rebound effects (emplacement, substitution, income, and macro). Doing so involves many terms and much complexity.

To date, visualizing the energy, expenditure, and consumption aspects of rebound phenomena has not been done in a numerically precise manner with a set of mutually consistent graphs. We introduce *rebound planes* to help advance clarity of (direct and indirect) rebound and adjustments (via emplacement, substitution, income, and macro effects) across all aspects (energy, expenditure, and consumption). Each aspect is represented by a path in its own plane, showing adjustments in response to the EEU.

Axes of the rebound planes represent direct and indirect effects, with direct effects shown on the x -axes, and indirect effects shown on the y -axes. Paths through energy, expenditure, and consumption planes consist of segments that represent changes due to the various rebound effects. Effects that include both direct and indirect rebound will show displacement along both axes and create a path in the x - y plane. See Section 3 for rebound planes for EEU examples of a car and an electric lamp and Appendix B for detailed mathematical descriptions for constructing paths on the rebound planes.

### 2.3. Software Tools

We developed an open source R package called ReboundTools to standardize and distribute the methods for calculating rebound magnitudes in our framework. ReboundTools can be found at https://github.com/MatthewHeun/ReboundTools. (See Heun [2023]). ReboundTools provides functions for (i) reading input data from a spreadsheet, (ii) performing rebound calculations, and (iii) generating rebound tables and rebound planes. ReboundTools was used for all calculations and all rebound planes in this paper.

To find the path to an example spreadsheet bundled with the package, users of ReboundTools can call the function ReboundTools::sample_eeu_data_path(). After filling the example spreadsheet with parameters for an EEU, users can call two functions (ReboundTools::load_eeu_data() and ReboundTools::rebound_analysis()) to perform all rebound calculations described in this paper. The function ReboundTools::path_graphs() creates rebound paths in the energy, expenditure, and consumption planes. Extensive documentation for ReboundTools can be found at https://matthewheun.github.io/ReboundTools/.

In addition, an Excel workbook that performs identical rebound calculations using the framework of this paper is available from University of Leeds at https://doi.org/10.5518/1634. (See Brockway, Heun and Semieniuk [2025]).

## 3. Results

In this section we present rebound calculation results for two examples: energy efficiency upgrades of a car (Section 3.1) and an electric lamp (Section 3.2). Univariate sensitivity studies for both examples (car and lamp) can be found in Appendix C.

### 3.1. Example 1: Purchase of a New Car

#### 3.1.1. Numerical Results: Car Example

**Figure 2.**
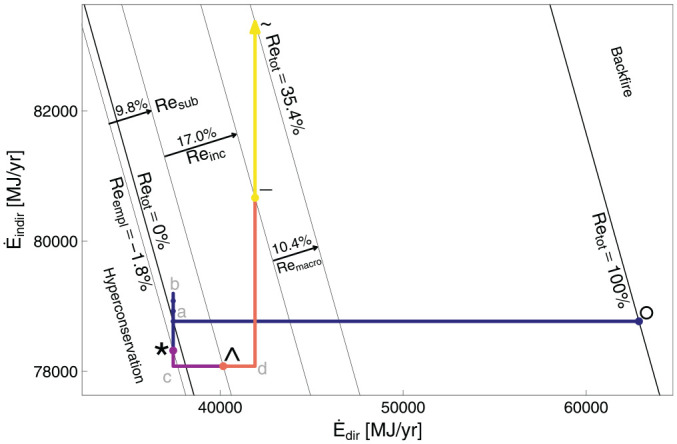
The energy plane for the car example. The macro factor, k=1 . *Note.* See Table 9 for meanings of path segments.

Armed with the data in Tables 1 to 3, and the equations in Section 2 of Part I, we calculate important values at each rebound stage, as shown in Table 7. Note that Table 7 applies to the car user. Across the macro effect (segment – 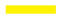 ~ in Figure 2), changes occur only in the macroeconomy. For the car user, no changes are recorded across the macro effect. Thus, the ~ (tilde) column is absent from Table 7. Rebound components for the car upgrade are shown in Table 8.

**Table 7.** Results for Car Example With Macro Factor ( k ) Assumed to be 1.

Quantity	Symbol [units]	Original (°)	After empl (*)	After sub (∧)	After inc (−)
Income rate	$\overset{\cdot }{M}$ [$/yr]	27,930			
Energy price	$p_{E}$ [$/MJ]	0.0208			
Device lifetime	$t_{\mathrm{life}}$ [yr]	14	14		
TVM factor (BOL)	$\tau_{\alpha }$ [–]	1.203	1.203		
TVM factor (EOL)	$\tau_{\omega }$ [–]	0.796	0.796		
Efficiency (engineering units)	η [mile/gal]	25	42		
Efficiency	η [mile/MJ]	0.197	0.332		
Energy service price	$p_{s}$ [$/mile]	0.105	0.063		
Embodied energy rate	${\dot{E}}_{emb}$ [MJ/yr]	2,429	2,857		
Capital cost rate	${\overset{\cdot }{C}}_{\mathrm{cap}}$ [$/yr]	2,533	2,518		
	$\tau_{\alpha }{\overset{\cdot }{C}}_{\mathrm{cap}}$ [$/yr]	3,048	3,030		
Ops. & maint. cost rate	${\overset{\cdot }{C}}_{\mathrm{OM}}$ [$/yr]	5,050	4,779		
Disposal cost	$C_{d}$ [$]	−300	−300		
Disposal cost rate	${\overset{\cdot }{C}}_{d}$ [$/yr]	−21	−21		
	$\tau_{\omega }{\overset{\cdot }{C}}_{d}$ [$/yr]	−17	−17		
Ops. & maint. and disposal cost rate	${\overset{\cdot }{C}}_{\mathrm{OMd}}$ [$/yr]	5,033	4,762		
Energy consumption rate	${\overset{\cdot }{E}}_{s}$ [MJ/yr]	62,885	37,432	40,167	41,903
Energy cost rate	${\overset{\cdot }{C}}_{s}$ [$/yr]	1,306	777	834	870
Net income rate	$\overset{\cdot }{N}$ [$/yr]	0	817	835	0
Energy service consumption rate	${\overset{\cdot }{q}}_{s}$ [mile/yr]		12,416	13,323	13,899
Other goods cost rate	${\overset{\cdot }{C}}_{g}$ [$/yr]		18,543	18,469	19,267

*Note.* There is no change for the consumer across the macro effect, so the last stage (~) is not shown. Blanks indicate unchanged values relative to previous or later values in the same row.

**Table 8.** Car Example: Rebound Results With Macro Factor ( k ) Assumed to be 1.

Rebound term	Value [%]
$Re_{\mathrm{dempl}}$	0.0
$Re_{\mathrm{emb}}$	1.7
$Re_{\mathrm{OMd}}$	−3.4
$Re_{\mathrm{dsub}}$	10.7
$Re_{\mathrm{isub}}$	−0.9
$Re_{\mathrm{dinc}}$	6.8
$Re_{\mathrm{iinc}}$	10.2
$Re_{\mathrm{macro}}$	10.4
$Re_{\mathrm{tot}}$	35.4

*Note.* See Section 4.1 in which we use an updated value of k=3 to obtain $Re_{tot}=56.2\%$ .

The emplacement effect has three components: the direct emplacement effect, the embodied energy effect, and the combined operations, maintenance, and disposal effects. Rebound from the direct emplacement effect ( $Re_{\mathrm{dempl}}$ ) is 0.0% always, because energy takeback (and, therefore, rebound) occurs after the upgraded device is emplaced. Indirect rebound due to the embodied energy effect ( $Re_{\mathrm{emb}}$ ) is 1.7%, due to the higher embodied energy rate ( $\Delta {\dot{E}}_{emb}^{*}=429$ MJ/yr) stemming from the electric battery in the hybrid EV car. Rebound due to the operations, maintenance, and disposal effects ( $Re_{\mathrm{OMd}}$ ) is small and negative (−3.4%), because of the slightly lower operations, maintenance, and disposal costs for the hybrid EV car.

The substitution effect has two components: direct and indirect substitution effect rebound. Rebound from direct substitution ( $Re_{\mathrm{dsub}}$ ) is positive, as expected (10.7%). The car user will, on average, prefer more driving purely from the change in relative prices because of the fuel economy enhancements (42 mpg > 25 mpg). In other words, due the relative price change, the more fuel-efficient car is driven 7.3% further each year. Conversely, the indirect substitution effect ( $Re_{\mathrm{isub}}$ ) is slightly negative (−0.9%) to achieve the same level of utility after increased driving. Indeed, across the substitution effect, less money is spent on other goods ( $\Delta {\hat{\overset{\cdot }{C}}}_{g}=-74$ $/yr). In Appendix C.7, we show how the displacement along an indifference curve alters the price elasticities, and in particular, that the uncompensated own price elasticity declines in magnitude. The decline slows the rate of additional consumption of energy-intensive driving, and attenuates the microeconomic rebound relative to assuming constant price elasticities.

The income effect also has two components: direct and indirect income effect rebound. The direct income effect ( $Re_{\mathrm{dinc}}$ ) is positive (6.8%), because the car user allocates some net savings to additional driving. Rebound from the indirect income effect ( $Re_{\mathrm{iinc}}$ ) is positive (10.2%) due to higher spending on other goods. Thus, the net savings after the substitution effect ( $\hat{\overset{\cdot }{N}}=835$ $/yr) translates into positive direct and indirect income rebound at the microeconomic level. Total microeconomic rebound (emplacement, substitution, and income effects) sums to $Re_{\mathrm{micro}}=25.0$ %.

Finally, in Part I we noted that the link between macroeconomic and microeconomic rebound is largely unexplored, so we assume a value of k=1 for both examples, initially. We return to the value for k in the Discussion (Section 4.1). With k assumed to be 1, the macro effect leads to macroeconomic rebound ( $Re_{\mathrm{macro}}$ ) of 10.4% for the car example, due to economic expansion caused by productivity enhancements arising from the more-efficient provision of the energy service (transportation).

#### 3.1.2. Rebound Visualizations: Car Example

Figure 2 shows the energy plane for the car example, assuming k=1 . The energy plane shows the direct energy consumption rate ( ${\overset{\cdot }{E}}_{\mathrm{dir}}$ ) on the x -axis and the indirect energy consumption rate ( ${\overset{\cdot }{E}}_{\mathrm{indir}}$ ) on the y -axis.^1^ Points °, *, ∧, −, and ~ represent stages between the rebound effects of Figure 1. Points a , b , c , and d represent intermediate stages. Table 9 provides the key for rebound path segments. Note that segment – 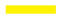 ~ appears only in the energy plane, because the framework tracks energy consumption but not expenditures or consumption for the macro effect.

**Table 9.** Segments in Rebound Planes.

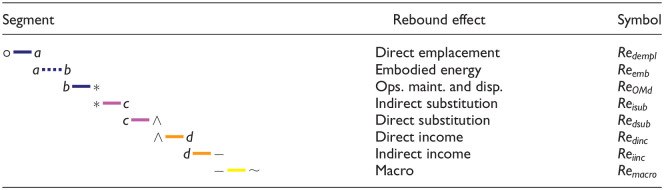

In the energy plane, lines with negative slope through points °, a , *, ∧, −, and ~ indicate energy consumption isoquants at key points. Point a lies on the $Re_{tot}=0\%$ line indicating that point a (and the $Re_{tot}=0\%$ line) is the point from which all rebound effects ( $Re_{\mathrm{empl}}$ , $Re_{\mathrm{sub}}$ , $Re_{\mathrm{inc}}$ , and $Re_{\mathrm{macro}}$ ) are measured. If rebound effects cause total energy demand to return to the original energy consumption level (negative sloping line through the ° point), all expected energy savings are taken back by rebound effects. Thus, the line of constant energy consumption through the ° point is labeled $Re_{tot}=100\%$ . The contribution of each rebound effect to total rebound is represented by the distance that each component’s segment moves across the rebound isoquants. Total rebound ( $Re_{\mathrm{tot}}$ ) is measured linearly between and beyond the $Re_{tot}=0\%$ and $Re_{tot}=100\%$ lines, with direct rebound in the x direction and indirect rebound in the y direction. The region below and to the left of the $Re_{tot}=0\%$ line exhibits negative rebound, indicating hyperconservation. The region above and to the right of the $Re_{tot}=100\%$ line shows backfire, that is, greater total energy consumption after the EEU than before it.

Segment * 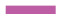 *c* moves in the negative y direction by definition of the indirect substitution effect, and segment *c* 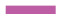 ^ moves in the positive x direction by the definition of the direct substitution effect. Both income effect segments (^ 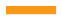 *d* and *d* 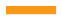
–) show more energy consumption, because net savings are spent on goods and services that rely on at least some energy consumption.^2^ Segment – 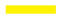 ~ always moves in the positive y direction, because macro effects lead to additional indirect energy consumption.

Note that rebound values from Table 8 are indicated on Figure 2 as sums of direct and indirect components for each effect: emplacement, substitution, income, and macro. Total rebound is also shown.

**Figure 3.**
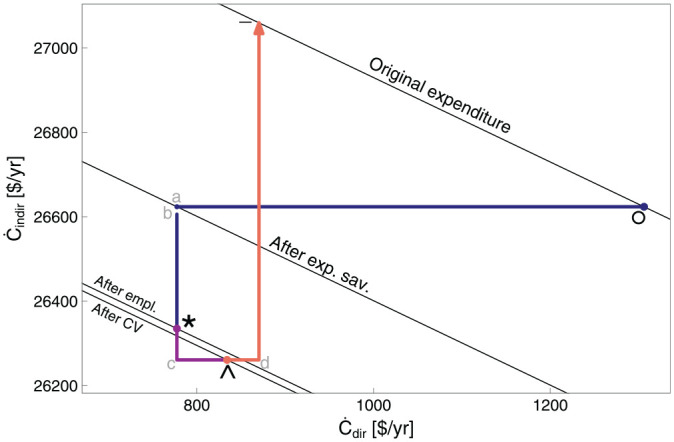
The expenditure plane for the car example. CV is compensating variation, the increase in consumption of the energy service and decrease in consumption of other goods and services to maintain constant utility. *Note.* See Table 9 for meanings of path segments.

Figure 3 shows the expenditure plane for the car example. The expenditure plane shows the direct expenditure rate on the energy service ( ${\overset{\cdot }{C}}_{\mathrm{dir}}$ ) on the x -axis and the indirect expenditure rate ( ${\overset{\cdot }{C}}_{\mathrm{indir}}$ , discounted when appropriate) on the y -axis. Lines with negative slope through points °, a , *, and ∧ indicate expenditure isoquants. The line through the ° point is an isoquant for the cost of purchasing the original consumption bundle at the original prices. The line through the * point is an isoquant for the cost of purchasing the original consumption bundle at the new prices. Segments *a*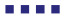 *b* and *b* 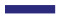 * could both move in the positive y direction, they could both move in the negative y direction, or they could move in opposite directions, depending on the results of the independent analyses for embodied energy and capital cost rates. The substitution effect along segments * 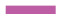 *c* and *c* 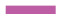 ^ will together, by definition, lead to lower expenditure due to the energy service price decline and the budget-reducing compensating variation (CV). The income effect (segments ^ 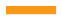 *d* and *d* 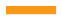 –) must bring expenditure back to the original expenditure line (equal to the budget constraint set by income in dollar or nominal terms) by assumptions about non-satiation and utility maximization in the device user’s decision function.

**Figure 4.**
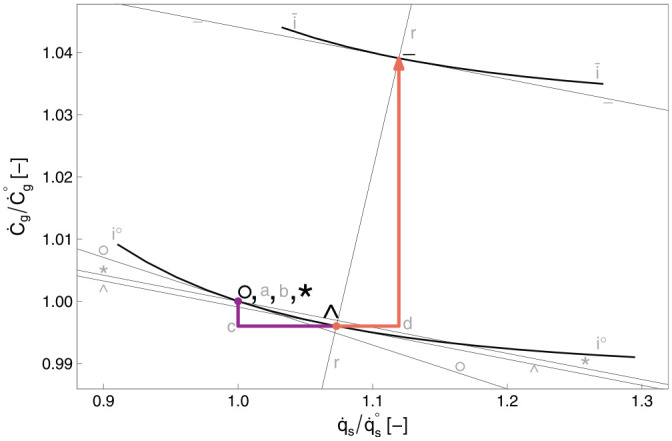
The consumption plane for the car example. *Note.* See Table 9 for meanings of path segments.

Figure 4 shows the consumption plane for the car example. The consumption plane shows the indexed rate of energy service consumption ( ${\overset{\cdot }{q}}_{s}/{\overset{\cdot }{q}}_{s}^{{}^{\circ}}$ ) on the x -axis and the indexed rate of other goods consumption ( ${\overset{\cdot }{C}}_{g}/{\overset{\cdot }{C}}_{g}^{{}^{\circ}}$ ) on the y -axis. Iso-expenditure loci of indexed energy service and other goods demand, that is budget constraints, are shown as lines with negative slope (lines °—°, *—*, ∧—∧, and −—−). Note that budget constraints °—° and −—− intersect at the y -axis (i.e., where x = 0), because the prices of other goods and services do not change as a result of the EEU. As defined in this framework, emplacement (by itself) does not alter consumption patterns, so the rate of energy service consumption and the rate of other goods consumption are unchanged across the emplacement effect ( ${\overset{\cdot }{q}}_{s}^{{}^{\circ}}={\overset{\cdot }{q}}_{s}^{*}$ and $\overset{.}{C_{g}^{{}^{\circ}}}=\overset{.}{C_{g}^{*}}$ , respectively). Thus, only movements after the * point are visible as a path in the consumption plane, and points °, *a*, *b*, and * collapse to the same location in the consumption plane.

Indifference curves for the CES utility model are denoted by $i^{{}^{\circ}}—$ $i^{{}^{\circ}}$ and $\bar{i}\;—$ $\bar{\text{i}}$ and represent lines of constant utility through the ° and − points. Prior to the EEU, the consumption basket (of the energy service and other goods) is represented by the ° point. The budget constraint, here in real terms, that is, the capacity to purchase either the energy service or other goods and services, is shown as isoquant °—°. The original budget constraint line (°_°) is tangent to the original indifference curve ( $i^{{}^{\circ}}—$ $i^{{}^{\circ}}$ ) at point °, the optimal consumption bundle prior to the EEU. The real budget line *—* indicates the (higher) capacity to purchase combinations of energy services and other goods and services using the same money needed to purchase the old consumption bundle but at the new, lower price for the energy service, thanks to the EEU.

The substitution effect leads to the cheaper, optimal CES-utility-preserving consumption bundle at the ∧ point. The substitution effect is shown by segments * 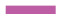 *c* (the indirect component, which represents the decrease in other goods consumption) and *c* 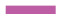 ^ (the direct component, which represents the increase in energy service consumption). Although the substitution effect is calculated in the consumption plane, its impact can be seen in the energy and expenditure planes.

In the consumption plane, the income expansion path under the CES utility model is a ray (r__r) from the origin through the ∧ point in the consumption plane. The pre- and post-income-effect points (∧ and −, respectively) lie along the r__r ray, due to homotheticity. The increased consumption rate of the energy service is represented by segment ^ 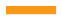 *d*, and the increased consumption rate of other goods and services is represented by segments *d* 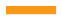 –.

Under non-homothetic utility models, the income expansion path will be closer to vertical in the consumption plane, as the device owner spends more net income ( $\hat{\overset{\cdot }{N}}$ ) on other goods and less on the energy service. In the limit, consumption of the energy service is already satiated, so net income is spent completely on other goods, resulting in a vertical income expansion path.

### 3.2. Example 2: Purchase of a New Electric Lamp

#### 3.2.1. Numerical Results: Lamp Example

With the data in Tables 4 to 6 and the equations in Section 2 of Part I in hand, we calculate important values at each rebound stage, as shown in Table 10. Rebound components for the lamp upgrade are shown in Table 11.

**Table 10.** Results for Lamp Example With Macro Factor ( k ) Assumed to be 1.

Quantity	Symbol [units]	Original (°)	After empl (*)	After sub (∧)	After inc (−)
Income rate	$\overset{\cdot }{M}$ [$/yr]	27,930			
Energy price	$p_{E}$ [$/MJ]	0.0358			
Device lifetime	$t_{\mathrm{life}}$ [yr]	2	10		
TVM factor (BOL)	$\tau_{\alpha }$ [–]	1.012	1.138		
TVM factor (EOL)	$\tau_{\omega }$ [–]	0.959	0.847		
Efficiency (engineering units)	η [lm hr/kW hr]	8,833	81,800		
Efficiency	η [lm hr/MJ]	2,454	22,722		
Energy service price	$p_{s}$ [$/lm hr]	0.00001457	0.00000157		
Embodied energy rate	${\overset{\cdot }{E}}_{\mathrm{emb}}$ [MJ/yr]	1.222	0.650		
Capital cost rate	${\overset{\cdot }{C}}_{\mathrm{cap}}$ [$/yr]	1.04	0.12		
	$\tau_{\alpha }{\overset{\cdot }{C}}_{\mathrm{cap}}$ [$/yr]	1.06	0.14		
Ops. & maint. cost rate	${\overset{\cdot }{C}}_{\mathrm{OM}}$ [$/yr]	0.00	0.00		
Disposal cost	$C_{d}$ [$]	0.00	0.00		
Disposal cost rate	${\overset{\cdot }{C}}_{d}$ [$/yr]	0.00	0.00		
	$\tau_{\omega }{\overset{\cdot }{C}}_{d}$ [$/yr]	0.00	0.00		
Ops. & maint. and disposal cost rate	${\overset{\cdot }{C}}_{\mathrm{OMd}}$ [$/yr]	0.00	0.00		
Energy consumption rate	${\overset{\cdot }{E}}_{s}$ [MJ/yr]	236.5	25.5	62.2	62.2
Energy cost rate	${\overset{\cdot }{C}}_{s}$ [$/yr]	8.46	0.91	2.22	2.22
Net income rate	$\overset{\cdot }{N}$ [$/yr]	0.00	8.46	11.30	0.00
Energy service consumption rate	${\overset{\cdot }{q}}_{s}$ [lm hr/yr]		580,350	1,412,867	1,413,439
Other goods cost rate	${\overset{\cdot }{C}}_{g}$ [$/yr]		27,920	27,916	27,927

*Note.* There is no change for the consumer across the macro effect, so the last stage (~) is not shown. Blanks indicate unchanged values relative to previous or later values in the same row.

**Table 11.** Lamp Example: Rebound Results With Macro Factor ( k ) Assumed to be 1.

Rebound term	Value [%]
$Re_{\mathrm{dempl}}$	0.0
$Re_{\mathrm{emb}}$	−0.3
$Re_{\mathrm{OMd}}$	0.0
$Re_{\mathrm{dsub}}$	17.4
$Re_{\mathrm{isub}}$	−6.4
$Re_{\mathrm{dinc}}$	0.0
$Re_{\mathrm{iinc}}$	17.3
$Re_{\mathrm{macro}}$	13.0
$Re_{\mathrm{tot}}$	41.1

*Note.* See Section 4.1 in which we use an updated value of k=3 to obtain $Re_{tot}=67.0\%$ .

The emplacement effect rebound components start with the direct emplacement effect ( $Re_{\mathrm{dempl}}$ ), which is always 0.0%. Indirect rebound due to the embodied energy effect ( $Re_{\mathrm{emb}}$ ) is −0.3%. Although the LED lamp has higher embodied energy ( $E_{\mathrm{emb}}^{*}=6.50$ MJ) than the incandescent lamp ( $E_{\mathrm{emb}}^{{}^{\circ}}=2.20$ MJ), the LED lamp has a much longer lifetime, meaning that the LED embodied energy *rate* ( ${\overset{\cdot }{E}}_{\mathrm{emb}}^{*}=0.65$ MJ/yr) is less than the incandescent embodied energy rate ( ${\overset{\cdot }{E}}_{\mathrm{emb}}^{{}^{\circ}}=1.22$ MJ/yr). Thus, the change in embodied energy rate ( $\Delta {\overset{\cdot }{E}}_{\mathrm{emb}}^{*}$ ) is −0.57 MJ/yr, and embodied energy rebound is negative ( $Re_{\mathrm{emb}}=-0.3$ %). Rebound due to the combined operations, maintenance, and disposal effects ( $Re_{\mathrm{OMd}}$ ) is 0.0%, because we assume no difference in operations, maintenance, or disposal costs between the incandescent lamp and the LED lamp.^3^

Direct substitution effect rebound ( $Re_{\mathrm{dsub}}$ ) is 17.4% due to the much higher LED lamp efficiency ( $\eta^{*}=81.8$ lm/W) compared to the incandescent lamp ( $\eta^{{}^{\circ}}=8.83$ lm/W), leading to increased demand for lighting (from ${\overset{\cdot }{q}}_{s}^{*}=580,350$ lmċhr/yr to ${\hat{\overset{\cdot }{q}}}_{s}=1,412,867$ lmċhr/yr) as shown by segment *c* 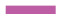 ^ in Figure 7. To maintain constant utility, consumption of other goods is reduced ( $\Delta {\hat{\overset{\cdot }{C}}}_{g}=-4.15$ /yr), yielding indirect substitution effect rebound ( $Re_{\mathrm{isub}}$ ) of −6.4%.

Income effect rebound arises from spending net energy cost savings associated with converting from the incandescent lamp to the LED lamp ( $\hat{\overset{\cdot }{N}}=11.30$ $/yr). Direct income effect rebound ( $Re_{\mathrm{dinc}}$ ) is 0.01%, positive but small, as the lamp user allocates some of the net savings to additional demand for lighting. The indirect income effect rebound is large ( $Re_{\mathrm{iinc}}=17.3$ %), due to the energy implications of increased spending on other goods. Total microeconomic level rebound (emplacement, substitution, and income effects) sums to $Re_{\mathrm{micro}}=28.1$ %.

Finally, macro effect rebound ( $Re_{\mathrm{macro}}$ ) is 13.0% with k assumed to be 1, due to economic expansion caused by productivity enhancements arising from the more-efficient provision of the energy service (lighting).

#### 3.2.2. Rebound Visualizations: Lamp Example

**Figure 5.**
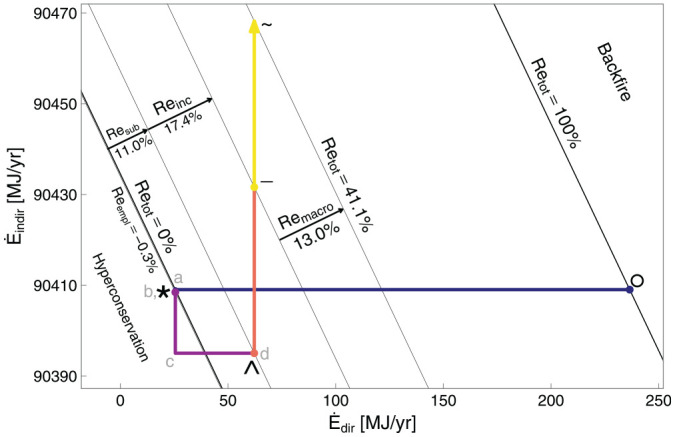
The energy plane for the lamp example. The macro factor, k=1 . *Note.* See Table 9 for meanings of path segments.

**Figure 6.**
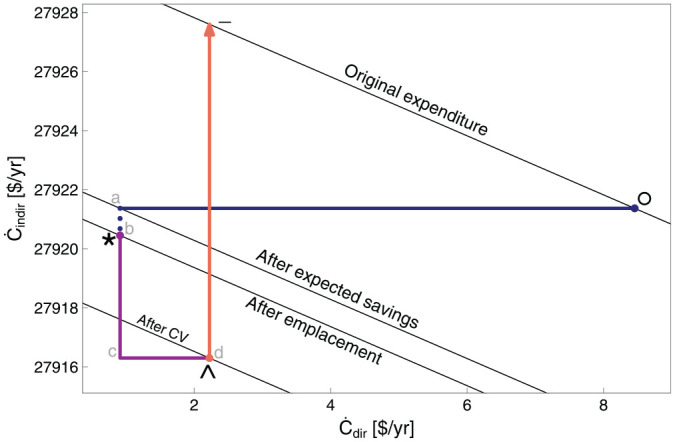
Expenditure plane for the lamp example. CV is compensating variation, the increase in consumption of the energy service and decrease in consumption of other goods and services to maintain constant utility. *Note.* See Table 9 for meanings of path segments.

**Figure 7.**
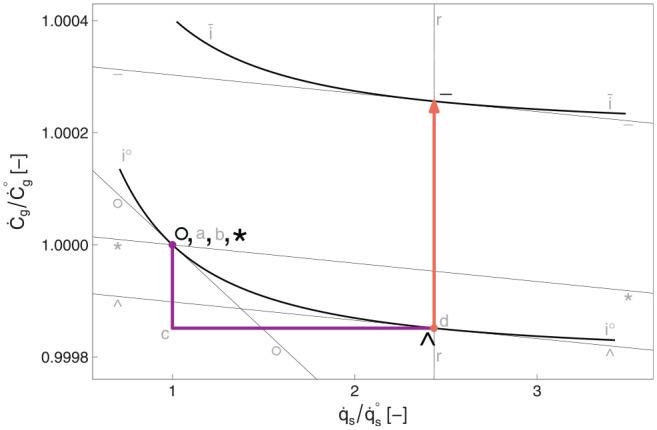
Consumption plane for the lamp example. *Note.* See Table 9 for meanings of path segments.

Figures 5 to 7 show energy, expenditure, and consumption planes for the lamp example.

## 4. Discussion

### 4.1. A First Attempt at Calculating Macro Rebound

Few previous studies explored the link between microeconomic and macroeconomic rebound. Inspired by Borenstein (2015) and others, the framework developed in Section 2 of Part I links macroeconomic rebound to microeconomic rebound via the macro factor ( k ) that scales magnitudes in the microeconomic portion of the framework. (See Section 2.5.4 of Part I.)

For the results presented in Section 3 above, we assumed a placeholder value of k=1 , meaning that every $1 of spending by the device user in the income effect generates only $1 of additional economic activity in the broader economy. There are no estimates of k , which ultimately traces the aggregate, long-run growth effects of a single, device-specific technical enhancement and is likely to differ between EEUs. However, using recent empirical estimates of sectoral multipliers we can ascertain ourselves that k should be different from 1 and choose a different value in line with those estimates.

Sectoral multipliers capture the impact of sectoral revenue increases into aggregate demand or GDP growth. While the idea of scale economies from larger markets for particular products have a long history in economic thought dating back at least to Smith (1776), data from input-output tables and recent advances in network theory allowed formalization of the spill-overs from sectoral to aggregate growth. First results from the literature show that three quarters of U.S. aggregate output growth originates from sectoral shocks, which are amplified through the production and investment network where one sector’s output is used as intermediate inputs and capital goods in others (Foerster et al. 2022). Durable goods are estimated by Foerster et al. to have the largest sectoral multiplier, and their effect on agggregate output is more than three times their sectoral growth. Since we are also considering durable goods, we adopt the value of k=3 to represent the long-run growth effect, fully aware that this can be only a first approximation.

After setting k=3 , we can recalculate all rebound components in our framework. Emplacement ( $Re_{\mathrm{empl}}$ ), substitution ( $Re_{\mathrm{sub}}$ ), and income ( $Re_{\mathrm{inc}}$ ) rebound magnitudes are unchanged after setting k=3 . However, we see that choosing a placeholder value of k=1 resulted in a low value for $Re_{\mathrm{macro}}$ and, therefore, $Re_{\mathrm{tot}}$ in Section 3. In Figures 2 and 5, the macro effect segments (– 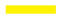 ~) should be three times longer than they appear. In Tables 8 and 11, the values of macro rebound ( $Re_{\mathrm{macro}}$ ) should triple to 31.2% and 39.0%, and the values of total rebound ( $Re_{\mathrm{tot}}$ ) should increase to 56.2% and 67.0% for the car and lamp examples, respectively. For the remainder of this paper, we use k=3 .

### 4.2. Comparison Between the Car and Lamp Case Studies

Tables 8 and 11 and selection of k=3 in Section 4.1 enable fuller comparisons between the car and lamp examples. Several points can be made.

First, the magnitude of every rebound effect is different between the two examples, the exception being direct emplacement rebound ( $Re_{\mathrm{dempl}}$ ) which is always 0.0% by definition. The implication is that every EEU needs to be analyzed separately. The magnitudes of the rebound effects for one EEU should never be assumed to apply to a different EEU.

Second, one cannot know a priori which rebound effects will be large and which will be small for a given EEU. Furthermore, some rebound effects are dependent upon economic parameters, such as energy intensity ( $I_{E}$ ). Thus, it is important to calculate the magnitude of all rebound effects for each EEU in each economy.

Third, the two examples illustrate the fact that embodied energy rebound ( $Re_{\mathrm{emb}}$ ) can be positive or negative, as discussed in Section 2.5.1 of Part I. The car’s embodied energy rebound is positive (1.7%) because of the high embodied energy of the hybrid’s battery relative to the internal combustion engine vehicle. Although the LED lamp’s embodied energy is larger than the incandescent lamp’s embodied energy, the LED lamp’s embodied energy rebound is small but negative (−0.3%), due to the longer life of the LED lamp compared to the incandescent lamp. Thus, each EEU should be analyzed independently for its embodied energy rebound.

Fourth, macro effect rebound is different between the two examples, owing to differences in net income ( ${\overset{\cdot }{N}}^{*}$ ) relative to expected savings ( ${\overset{\cdot }{S}}_{\mathrm{dev}}$ ). (For the car, $Re_{\mathrm{macro}}$ is 31.2%. For the lamp, $Re_{\mathrm{macro}}$ is 39.0%). The efficiency gain for the lamp is far greater than the efficiency gain for the car, leading to much different rates of net income ( ${\overset{\cdot }{N}}^{*}$ ) and different macro rebound values.

### 4.3. Comparison to Previous Rebound Estimates

Tables 12 and 13 compare car and lamp results (with k=3 ) to results from previous studies. The suite of comparison studies is neither comprehensive nor definitive of car and lamp EEUs; rather, they are examples that show the sort of calculations and estimations carried out in the general literature using a variety of methods. That said, many of the studies are highly cited, thereby carrying sufficient academic weight for our purposes. Tables 12 and 13 and their associated references enable two types of observations, comparing (i) coverage of rebound components and (ii) magnitudes and associated calculation or estimation methods.

**Table 12.** Rebound Magnitude Comparisons for the Car Example. All Numbers in %. k=3 is Assumed.

Rebound study	Coverage	Analysis method	$Re_{\mathrm{micro}}$			$Re_{\mathrm{macro}}$	$Re_{\mathrm{dir}}$	$Re_{\mathrm{indir}}$	$Re_{\mathrm{tot}}$
			$Re_{\mathrm{empl}}$	$Re_{\mathrm{sub}}$	$Re_{\mathrm{inc}}$				
This paper (2024)	U.S., 2020	Energy, expenditure, and consumption planes	-1.8	9.8	17.0	31.2	17.6	38.6	56.2
Small and Van Dender (2007)	U.S., 1967–2001	Elasticity of VMT w.r.t.fuel cost per mile					4.5 (short run, 1967–2001) 22.2 (long run, 1967–2001) 2.2 (short run, 1997–2001)10.7 (long run, 1997–2001)		
Greene (2012)	U.S., 1966–2007	Elasticities of transport fuel w.r.t. price and efficiency					4 (short run) 16 (long run)		
Koesler (2013)	Germany, 2009	Static CGE model, 10% efficiency shock					≤64	≤16	56
Thomas and Azevedo (2013)	U.S., 2004	Expenditure/cross price elasticities of personal transport fuels, using household spending survey data					10	6	
Borenstein (2015)	U.S., 2012	Microeconomic framework		13 (6–28)	11				
Chitnis and Sorrell (2015)	UK, 1964–2014	Estimated own/cross price elasticities of transport fuels, uses household spending survey data		72	5		55	23	86
Gillingham, Jenn and Azevedo (2015)	Pennsylvania, 2000–2010	Estimation of gasoline price elasticity of driving demand, from dataset of 75 million vehicle inspection records, including odometer data					10 (short run)		
Stapleton, Sorrell and Schwanen (2016)	UK 1970–2011	Elasticity of VMT w.r.t. fuel cost/prices					9–36		
Moshiri and Aliyev (2017)	Canada, 1997–2009	Price elasticity of transport fuel, using household spending survey data					82–88		
Duarte, Sánchez-Chóliza and Sarasa (2018)	Spain, 2010–2030	Dynamic CGE model, efficiency shock							26 (short run) 52 (long run)

Note: $Re_{\mathrm{tot}}=Re_{\mathrm{empl}}+Re_{\mathrm{sub}}+Re_{\mathrm{inc}}+Re_{\mathrm{macro}}$ , $Re_{\mathrm{tot}}=Re_{\mathrm{micro}}+Re_{\mathrm{macro}}$ , and $Re_{\mathrm{tot}}=Re_{\mathrm{dir}}+Re_{\mathrm{indir}}$ .

**Table 13.** Rebound Magnitude Comparisons for the Lamp Example. All Numbers in %. k=3 is Assumed.

Rebound study	Coverage	Analysis method	$Re_{\mathrm{micro}}$			$Re_{\mathrm{macro}}$	$Re_{\mathrm{dir}}$	$Re_{\mathrm{indir}}$	$Re_{\mathrm{tot}}$
			$Re_{\mathrm{empl}}$	$Re_{\mathrm{sub}}$	$Re_{\mathrm{inc}}$				
This paper (2024)	U.S., 2020	Energy, expenditure, and consumption planes	-0.3	11.0	17.4	39.0	17.4	49.7	67.0
Guertin, Kumbhakar and Duraiappah (2003)	Canada, 1993	Econometric residential energy demand model based on Canadian house-hold data					32–49		
Freire-González (2011)	Catalonia, Spain, 2000–2008	Input-output based energy model, utilizing expenditure/cross price elasticities					49	16	
Thomas and Azevedo (2013)	U.S., 2004	Expenditure/cross price elasticities of home electricity use, using household spending survey data					10	10	
Schleich, Mills and Dütschke (2014)	Germany, 2012	Survey of electricity consumption in 6,409 German households					6		
Borenstein (2015)	U.S., 2012	Microeconomic framework		14 (6–37)	6				
Chitnis and Sorrell (2015)	UK, 1964–2014	Estimated own/cross price elasticities of transport fuels, uses household spending survey data		14	35		41	8	49
Duarte, Sánchez-Chóliza and Sarasa (2018)	Spain, 2010–2030	Dynamic CGE model, efficiency shock							12 (short run) 51 (long run)
Barkhordar (2019)	Iran, 2018–2040	Dynamic CGE model					28 (average)		43 (average)
Chitnis, Fouquet and Sorrell (2020)	UK, 1964–2015	Household demand analysis via Linear approximation to the Almost Ideal Demand System (LAIDS)					95	-41	54
Shojaeddini and Gilbert (2022)	U.S., 2009	Price elasticity of lighting from cross sectional data from the 2009 Residential Energy Consumption Survey (RECS)					18–29		

Note: $Re_{\mathrm{tot}}=Re_{\mathrm{empl}}+Re_{\mathrm{sub}}+Re_{\mathrm{inc}}+Re_{\mathrm{macro}}$ , $Re_{\mathrm{tot}}=Re_{\mathrm{micro}}+Re_{\mathrm{macro}}$ , and $Re_{\mathrm{tot}}=Re_{\mathrm{dir}}+Re_{\mathrm{indir}}$ .

First, we see that none of the comparison studies report all rebound effects considered in this paper. Also, no previous studies report either emplacement rebound ( $Re_{\mathrm{empl}}=Re_{\mathrm{emb}}+Re_{\mathrm{OMd}}$ ) or include all of direct and indirect, substitution and income microeconomic rebound effect combinations. In addition, none of the other studies report macro rebound ( $Re_{\mathrm{macro}}$ ) by itself. In fact, only four and five of the ten studies in each category (car and lamp, respectively) report total rebound ( $Re_{\mathrm{tot}}$ ). Therefore, by carefully including all rebound components in the framework and elucidating all rebound components in Part II, we are (i) helping to advance conceptual clarity in the field of energy rebound, which (ii) may enable future studies to estimate a broader range of rebound components.

We also observe that studies which provide total rebound are based on a top-down calculation of overall, economy-wide rebound, rather than the bottom-up “sum-of-components” approach that we employ. That finding is instructive. It supports the view that a rigorous analysis framework that sets out individual rebound components has been missing, which informed the objective for Part I of this paper. Further, the finding means that comparisons between top-down estimations or calculations of total, economy-wide rebound may also be of limited value, because the rebound effects included or excluded may not be clear, giving an appearance of a “black box” calculation approach.^4^

Second, helpful insights can be gained from comparison of rebound magnitudes and calculation methods. Greatest alignment between our values and earlier values appears within the direct (microeconomic) rebound ( $Re_{\mathrm{dir}}$ ) column in Tables 12 and 13. Our car (17.6%) and lamp (17.4%) values are in the lower half of the comparison studies for both cases (10% to 49% for the car and 10% to 55% for the lamp). This direct rebound alignment may be due to the easier determination of direct rebound, from either empirical data (e.g., Small and Van Dender [2007]) or via own price elasticities (e.g., Chitnis and Sorrell [2015]).^5^

For indirect rebound ( $Re_{\mathrm{indir}}$ ), there is little agreement on the magnitude of rebound effects. Our values for car (38.6%) and lamp (49.7%) indirect rebound magnitudes are higher than those found in the comparison studies for either the car (6% to 23%) or the lamp (8% to 16%) cases. The most likely cause of our larger indirect rebound values is that we include both micro and macro rebound levels, whereas the comparison studies focus mainly on microeconomic rebound only (commonly via cross price elasticities). In other words, comparisons of our indirect rebound values with the studies in Tables 12 and 13 may be too simple and not very meaningful, as we (alone) include macro-level effects in indirect rebound. If we exclude $Re_{\mathrm{macro}}$ from $Re_{\mathrm{indir}}$ , our indirect microeconomic rebound values become 7.5% (car) and 10.7% (lamp), which fit within the ranges reported by the car (6% to 23%) and lamp (−41% to 16%) comparison studies.

For total rebound ( $Re_{\mathrm{tot}}$ ), our values of 56.2% (car) and 67.0% (lamp) are close to those in the comparison studies for both the car (49% to 51%) and lamp (43% to 51%) examples. Beyond that, comparisons (as noted earlier) are inhibited by methodological differences between previous studies (top-down methods) and our bottom-up approach for calculating total rebound.

### 4.4. Sensitivity of Rebound to $\varepsilon_{{\overset{\cdot }{q}}_{s},p_{s}}^{{}^{\circ}}$

The effect of the uncompensated own price elasticity of energy service consumption ( $\varepsilon_{{\overset{\cdot }{q}}_{s},p_{s}}^{{}^{\circ}}$ ) on rebound deserves additional consideration, because it dictates the magnitude of direct substitution rebound and thereby affects other rebound components that follow. $\varepsilon_{{\overset{\cdot }{q}}_{s},p_{s}\;}^{{}^{\circ}}$ is important, because it models device owner behavior with respect to additional consumption of the energy service. As $\varepsilon_{{\overset{\cdot }{q}}_{s},p_{s}}^{{}^{\circ}}$ becomes more negative, the device owner increases consumption of the energy service after the EEU. We illustrate with the lamp example.

Because the microeconomic portion of this framework is focused on adjustments caused by a single EEU (in this example, replacing a single incandescent lamp with a single LED), $\varepsilon_{{\overset{\cdot }{q}}_{s},p_{s}}^{{}^{\circ}}$ should account for only the behavioral adjustment of using a lamp for more hours per day. The device owner installing additional lamps with the savings generated by the EEU would fall under the income effect in our framework. Installing additional lamps elsewhere in the economy would fall under the macro effect.

Fouquet and Pearson (2011, Table 3) estimate $\varepsilon_{{\overset{\cdot }{q}}_{s},p_{s}}^{{}^{\circ}}=-0.6$ for the most recent decade of their study (2000–2010). As with most historical lighting rebound studies, the Fouquet and Pearson estimate applies to the whole economy and will include emplacement of additional lamps. Thus, the value $\varepsilon_{{\overset{\cdot }{q}}_{s},p_{s}}^{{}^{\circ}}=-0.6$ is not entirely applicable to our framework, which is focused on single-device replacement. The value applicable to single-device replacements (and appropriate for this framework) is expected to be less negative. Like us, Borenstein (2015) focuses on single-device replacements and uses a range: $-0.8<\varepsilon_{{\overset{\cdot }{q}}_{s},p_{s}}^{{}^{\circ}}<-0.4$ . We select $\varepsilon_{{\overset{\cdot }{q}}_{s},p_{s}}^{{}^{\circ}}=-0.4$ (Table 6), acknowledging that the single-device elasticity is expected to be less negative than the economy-wide value from Fouquet and Pearson ( −0.6 ). With $\varepsilon_{{\overset{\cdot }{q}}_{s},p_{s}}^{{}^{\circ}}=-0.4$ , Table 13 indicates that consumption of illumination from the single lamp increases by a factor of 2.4 from 3.0 hr/day originally to 7.3 hr/day after the substitution effect. This may mean that the more efficient lamp isn’t switched off when the device owner leaves a room or the house for a period of time.

To our knowledge, the only study that focuses on single-device rebound and differentiates between “burn time” rebound and “luminosity” rebound is Schleich, Mills and Dütschke (2014). Their methodology to determine burn time rebound relies on surveys and self-reported estimates rather than in-home measurements of the additional burn time per day for an LED lamp compared to an incandescent lamp. Because of this shortcoming, we prefer the value of $\varepsilon_{{\overset{\cdot }{q}}_{s},p_{s}}^{{}^{\circ}}=-0.4$ , as motivated above.

Regardless, Schleich, Mills and Dütschke (2014) and Fouquet and Pearson (2011) can be used to assess the sensitivity of total rebound to the value of $\varepsilon_{{\overset{\cdot }{q}}_{s},p_{s}}^{{}^{\circ}}$ . Indeed, a value for $\varepsilon_{{\overset{\cdot }{q}}_{s},p_{s}}^{{}^{\circ}}$ can be back-calculated from the estimate of burn time rebound, given as 4% (Schleich, Mills and Dütschke 2014, 40, Table 2, “All bulbs, IL to LED” row). Burn time rebound is equivalent to our direct substitution rebound ( $Re_{\mathrm{dsub}}$ ) and implies a value for uncompensated own price elasticity of energy service consumption of $\varepsilon_{{\overset{\cdot }{q}}_{s},p_{s}}^{{}^{\circ}}=-0.13$ .^6^ Our value of $\varepsilon_{{\overset{\cdot }{q}}_{s},p_{s}}^{{}^{\circ}}=-0.4$ lies between Fouquet and Pearson ( −0.6 ) and the implied elasticity from Schleich et al. ( −0.13 ).

**Figure 8.**
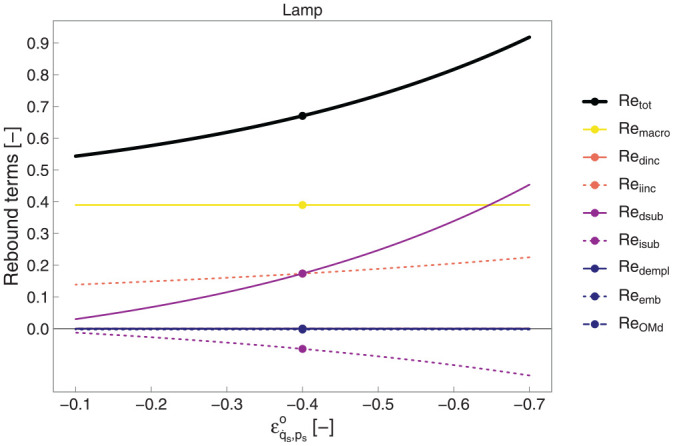
Sensitivity of rebound components to uncompensated own price elasticity of energy service demand ( $\varepsilon_{{\overset{\cdot }{q}}_{s},p_{s}}^{{}^{\circ}}$ ) in the lamp example. The macro factor, k=3 . *Note.* The lines for $Re_{\mathrm{dempl}}$ , $Re_{\mathrm{dinc}}$ , $Re_{\mathrm{emb}}$ , and $Re_{\mathrm{OMd}}$ are nearly coincident. Reversed x -axis scale.

Figure 8 shows the univariate sensitivity to $\varepsilon_{{\overset{\cdot }{q}}_{s},p_{s}\;}^{{}^{\circ}}$ for the lamp example and enables estimation of total rebound in our framework using the implied elasticity from Schleich, Mills and Dütschke (2014) and the recent value from Fouquet and Pearson (2011). (See Appendix C for additional univariate sensitivity analyses.) Specifically, total rebound for the lamp example is 55.2 % (with $\varepsilon_{{\overset{\cdot }{q}}_{s},p_{s}}^{{}^{\circ}}=-0.13$ , the implied value from Schleich et al.), 67.0 % (with $\varepsilon_{{\overset{\cdot }{q}}_{s},p_{s}}^{{}^{\circ}}=-0.4$ , our preferred value), or 81.7 % (with $\varepsilon_{{\overset{\cdot }{q}}_{s},p_{s}}^{{}^{\circ}}=-0.6$ , from Fouquet and Pearson).

### 4.5. Comparison of CES With Satiated and Constant Price Elasticity (CPE) Utility Models

In Section 2.5.3 of Part I, we showed income-effect rebound expressions under the limiting condition of already-satiated consumption of the energy service such that the income expansion path is a vertical line in the consumption plane of Figures 4 and 7. Here, we discuss the numerical impact of the different utility models.

Table 14 compares income-effect rebound under the CES utility model, the bounding condition of satiated consumption of the energy service, and the constant price elasticity (CPE) utility model.^7^

**Table 14.** Comparison of Substitution Effects ( $Re_{\mathrm{dsub}}$ , $Re_{\mathrm{isub}}$ , $Re_{\mathrm{sub}}$ ), Income Effects, ( $Re_{\mathrm{dinc}}$ , $Re_{\mathrm{iinc}}$ , and $Re_{\mathrm{inc}}$ ), and Total ( $Re_{\mathrm{tot}}$ ) Rebound for the CES Utility Model, Satiated Consumption of the Energy Service, and the CPE Utility Model for Both Car and Lamp Examples.

Rebound term	Car example			Lamp example		
	CES	Satiated	CPE	CES	Satiated	CPE
$Re_{\mathrm{dsub}}$ [%]	10.7	10.7		17.37	17.37	
$Re_{\mathrm{isub}}$ [%]	−0.9	−0.9		−6.37	−6.37	
$Re_{\mathrm{sub}}$ [%]	9.8	9.8	15.0	11.00	11.00	15.37
$Re_{\mathrm{dinc}}$ [%]	6.8	0.0		0.01	0.00	
$Re_{\mathrm{iinc}}$ [%]	10.2	10.6		17.35	17.35	
$Re_{\mathrm{inc}}$ [%]	17.0	10.6	10.4	17.36	17.35	12.99
$Re_{\mathrm{tot}}$ [%]	56.2	49.8		67.04	67.03	

In the car example, income effect rebound ( $Re_{\mathrm{inc}}$ ) reduces from 17.0% to 10.6% when moving from the CES utility model to the bounding condition of already-satiated consumption of the energy service. Total rebound ( $Re_{\mathrm{tot}}$ ) goes from 56.2% to 49.8%. On the other hand, the lamp example shows negligible change in total rebound ( $Re_{\mathrm{tot}}$ ), moving from 67.04% to 67.03%.

The reason for the nearly unchanged value for total rebound ( $Re_{\mathrm{tot}}$ ) in the lamp example is evident in the consumption plane of Figure 7. In the CES (homothetic) utility model shown in Figure 7, there is almost no income-effect spending on more of the energy service. Almost all spending of net income ( $\hat{\overset{\cdot }{N}}$ ) is on other goods. The path between the ∧ and − points is nearly vertical already. In contrast, the path from ∧ to − in the car example (Figure 4) is decidedly *not* vertical and a reduction in income-effect rebound ( $Re_{\mathrm{inc}}$ ) is observed when moving from the CES utility model to the bounding condition of already satiated energy service consumption. Reality is probably somewhere in between.

Calculation of substitution rebound under the constant price elasticity (CPE) utility model, which approximates the substitution and income effects using only the uncompensated own price elasticity of energy service consumption ( $\varepsilon_{{\overset{\cdot }{q}}_{s},p_{s}}^{{}^{\circ}}$ ), systematically overestimates substitution effect rebound and underestimates income effect rebound. That’s by construction since income, not utility, is held constant when calculating substitution of the energy service for other goods consumption with the CPE model. And the income effect, in the CPE utility model, allows spending on other goods only, which leads to a lower income rebound than in the satiated model, since the absence of compensating variation leaves less income to spend. Once again, the specific case determines the deviation of the sum of substitution and income rebound from an exact (in our case, CES) model. Table 14 shows that while the sum of substitution and income effects is 1.4 percentage points smaller for the CPE utility model relative to the CES utility model, they are nearly the same in the lamp example.

### 4.6. Energy Price Rebound

Section 3.2, equation (36), and Appendix F of Part I provide an extension to the framework involving energy price rebound ( $Re_{p_{E}}$ ). This section quantifies energy price rebound for the car and lamp examples.

To quantify energy price rebound, data are needed for personal consumption ( ${\overset{\cdot }{E}}^{{}^{\circ}}$ ) of the type of energy used by the device, including energy for the upgraded device and all other devices. For the car example, there is typically little other household gasoline consumption besides for cars, so we assume ${\overset{\cdot }{E}}^{{}^{\circ}}$ equal to ${\overset{\cdot }{E}}_{s}^{{}^{\circ}}/0.95$ . For the lamp example, a median U.S. household consumes about 10,000 kWċhr/yr of electricity (U.S. Energy Information Agency 2023). Given that there are 2.5 persons per U.S. household (Statista 2024), an individual consumes electricity at a rate of ${\overset{\cdot }{E}}^{{}^{\circ}}=4000$ kWċhr/yr.

We also need data for the price elasticity of energy supply ( $\varepsilon_{{\overset{\cdot }{Q}}_{E},p_{E}}$ ). For the car case, we take the price elasticity of gasoline supply to be 0.29 from Coyle, DeBacker and Prisinzano (2012). For the lamp case, we adopt the value of 0.33 from Ghoddusia and Roy (2017, Table 3).

**Figure 9.**
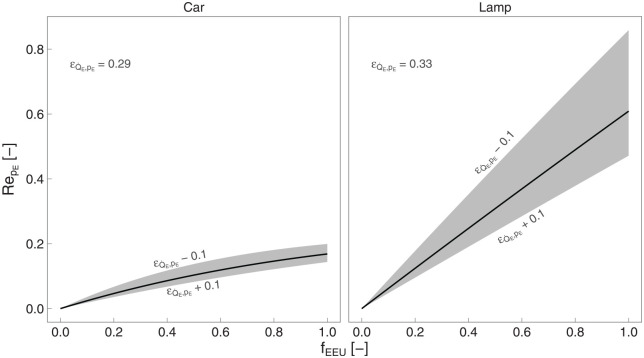
Energy price rebound ( $Re_{p_{E}}$ ) as a function of the fraction of all devices replaced by higher-efficiency versions ( $f_{\mathrm{EEU}}$ ). *Note.* Black lines represent the nominal energy price elasticity of energy supply ( $\varepsilon_{{\overset{\cdot }{Q}}_{E},p_{E}}$ ). Gray bands provide ±0.1 range in $\varepsilon_{{\overset{\cdot }{Q}}_{E},p_{E}}$ .

Parameterizing on the fraction of all devices in the economy that are upgraded ( $f_{\mathrm{EEU}}$ ) and the energy price elasticity of energy supply ( $\varepsilon_{{\overset{\cdot }{Q}}_{E},p_{E}}$ ) yields Figure 9. As expected, price-effect rebound ( $Re_{p_{E}}$ ) grows as more devices are upgraded, that is, as $f_{\mathrm{EEU}}$ increases. Furthermore, inelastic energy supply (smaller $\varepsilon_{{\overset{\cdot }{Q}}_{E},p_{E}}$ ) leads to higher price-effect rebound.

In these examples, the car upgrade yields little additional freed cash beyond the (slightly) cheaper fuel for the car, so there is limited spending on other goods and services and little additional indirect energy demand. In contrast, the upgrade of the electric lamp is much more likely to provide energy price rebound, because electricity for the upgraded lamp is a small fraction of total electricity consumption by the consumer. *All* electricity purchased by the consumer becomes cheaper when the price of electricity falls due to widespread lamp upgrades throughout the economy, leading to freed cash spent on other goods and services which, themselves, demand energy at the energy intensity of the economy.

At 100% penetration of LED lamps ( $f_{\mathrm{EEU}}=1$ ) and at the nominal energy price elasticity of supply ( $\varepsilon_{{\overset{\cdot }{Q}}_{E},p_{E}}=0.33$ ), energy price rebound is $Re_{p_{E}}=60.9$ %. Combined with consumer sided rebound of 67.0% from Section 4.1, the sum of consumer-sided and supply-side rebound is 127.9%, demonstrating that backfire could occur under conditions of full penetration of the lamp EEU.

## 5. Conclusions

In this paper (Part II of two), we help to advance clarity in the field of energy rebound by (i) developing mutually consistent and numerically precise visualizations of rebound effects in energy, expenditure, and consumption planes, (ii) operationalizing the macro factor, (iii) documenting in detail new calculations of rebound for car and lighting upgrades, (iv) showing the extensibility of our framework by applying it to estimate energy price rebound, and (v) providing information about new open source software tools for calculating and visualizing rebound for any energy efficiency upgrade. We encourage energy analysts and economists to use visualizations like the energy, expenditure, and consumption planes to document and visualize rebound calculations going forward. Our hope is that additional clarity will (i) narrow the gap between economists and energy analysts, (ii) lead to deeper interdisciplinary understanding of rebound phenomena, and (iii) enable energy and climate policy that takes fuller account of rebound.

From the application of the framework in Part II, we draw two important conclusions. First, the car and lamp examples (Section 3) show that the framework enables quantification of rebound magnitudes at microeconomic and macroeconomic levels, including energy, expenditure, and consumption aspects of direct and indirect rebound for emplacement, substitution, income, and macro effects. Second, the examples show that magnitudes of all rebound effects vary with the type of EEU performed. Thus, values for rebound effects for one EEU should never be assumed to apply to a different EEU, and it is important to calculate the magnitude of all rebound effects for each EEU in each economy.

Further work could be pursued in several areas. (i) Additional empirical studies could be performed to calculate the magnitude of different rebound effects for a variety of real-life EEUs. (ii) Deeper study of macro rebound is needed, including improved determination of the value of the macro factor ( k ). (iii) The framework could be used to study the distribution of rebound values across socioeconomic and demographic groups (Carroll et al. 2017). (iv) The rebound effects of fossil-energy taxes could be studied, especially for the web of interconnected dynamic effects among rebound components that are functions of the energy intensity of the economy ( $I_{E}$ ). (v) Sensitivities of rebound components to model parameters could be investigated more fully than in Appendix C, although this will be challenging work because many rebound parameters are covariant. For example, post-EEU efficiency ( $\eta^{*}$ ) is unlikely to be independent of post-EEU capital cost ( $C_{\mathrm{cap}}^{*}$ ). (vi) The framework could be extended to encompass fuel-switching EEUs, such as the move from a gasoline car to an all-electric car. (vii) This framework could be embedded in energy-economy models to better include rebound effects in discussions of macro energy modeling, energy policy, and CO_2_ emissions mitigation.

## Figures and Tables

**Figure C.1.**
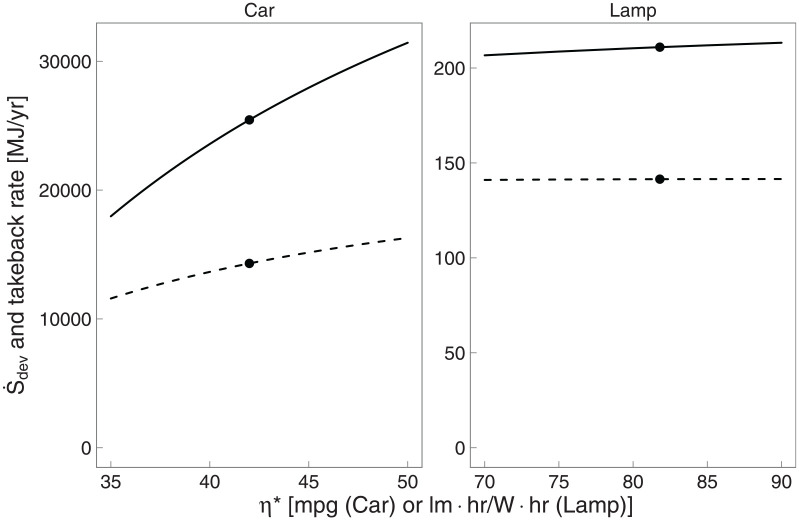
Expected energy savings rate ( ${\overset{\cdot }{S}}_{\mathrm{dev}}$ , solid line) and takeback rate (dashed line) sensitivity to post-EEU efficiency ( $\eta^{*}$ ). The macro factor, k=3 . *Note.* Different x - and y -axis scales.

**Figure C.2.**
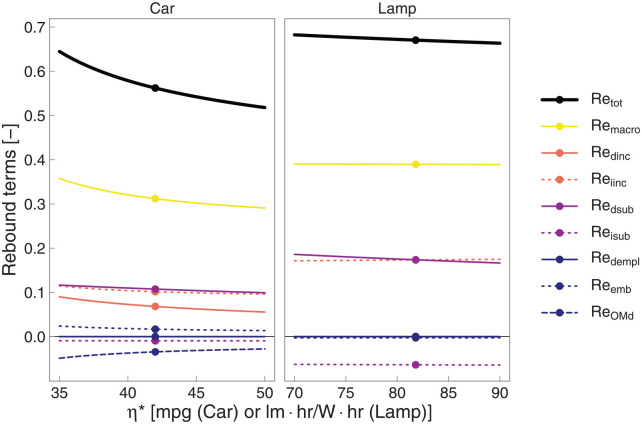
Sensitivity of rebound components to post-EEU efficiency ( $\eta^{*}$ ). The macro factor, k=3 .

**Figure C.3.**
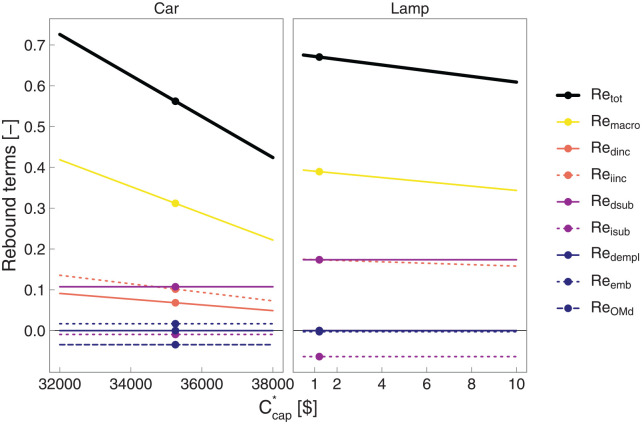
Sensitivity of rebound components to capital cost ( $C_{\mathrm{cap}}^{*}$ ). The macro factor, k=3 .

**Figure C.4.**
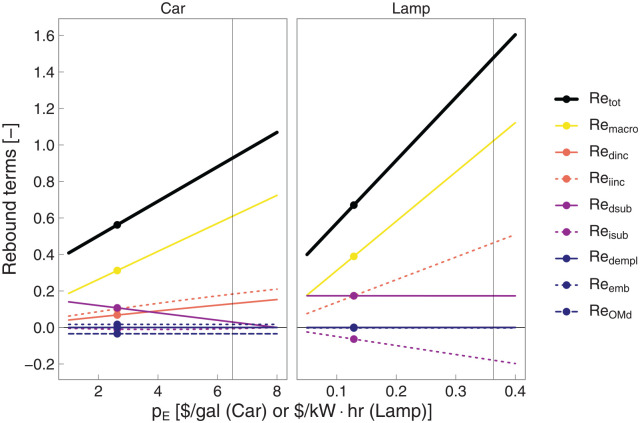
Sensitivity of rebound components to energy price ( $p_{E}$ ). German energy prices denoted by vertical lines. The macro factor, k=3 .

**Figure C.5.**
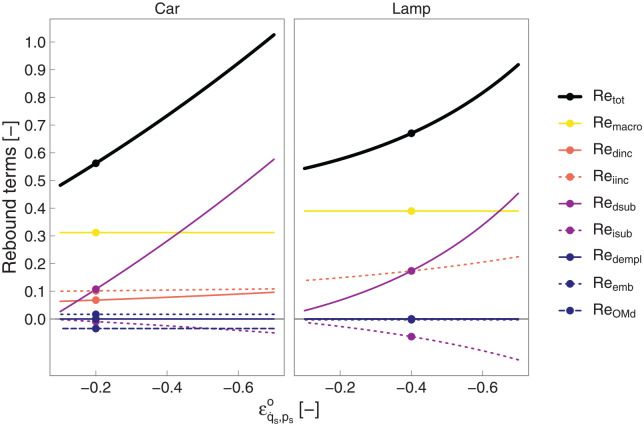
Sensitivity of rebound components to uncompensated own price elasticity of energy service demand ( $\varepsilon_{{\overset{\cdot }{q}}_{s},p_{s}}^{{}^{\circ}}$ ). The macro factor, k=3 . The lamp portion of this figure appears as Figure 8. *Note*. Reversed x -axis scale.

**Figure C.6.**
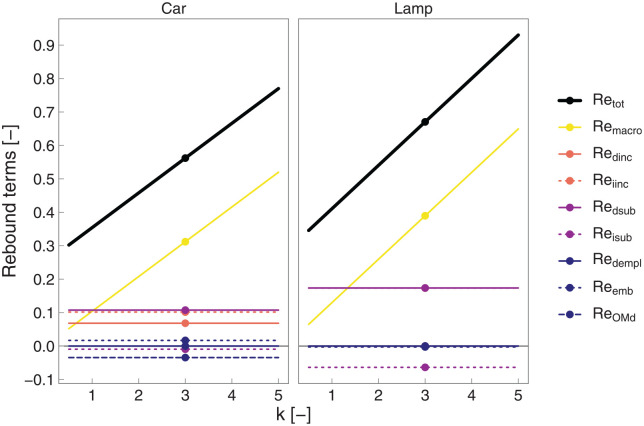
Sensitivity of rebound components to the macro factor ( k ).

**Figure C.7.**
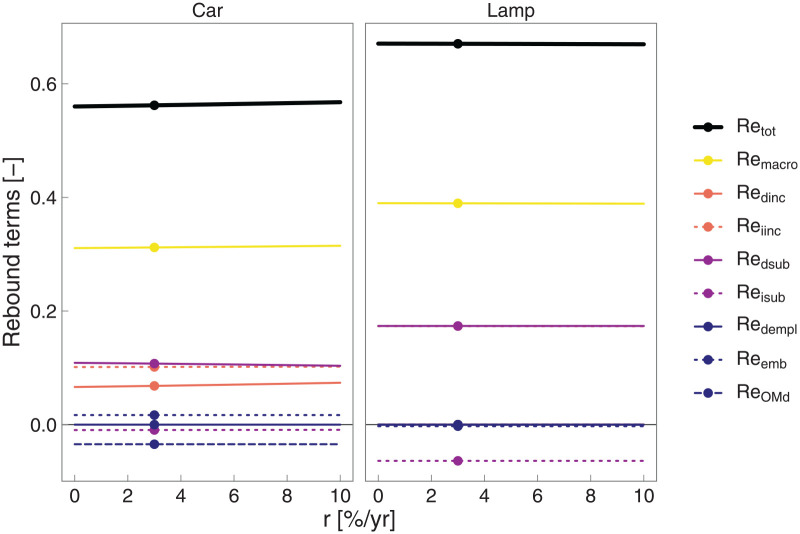
Sensitivity of rebound components to discount rate ( r ).

**Figure C.8.**
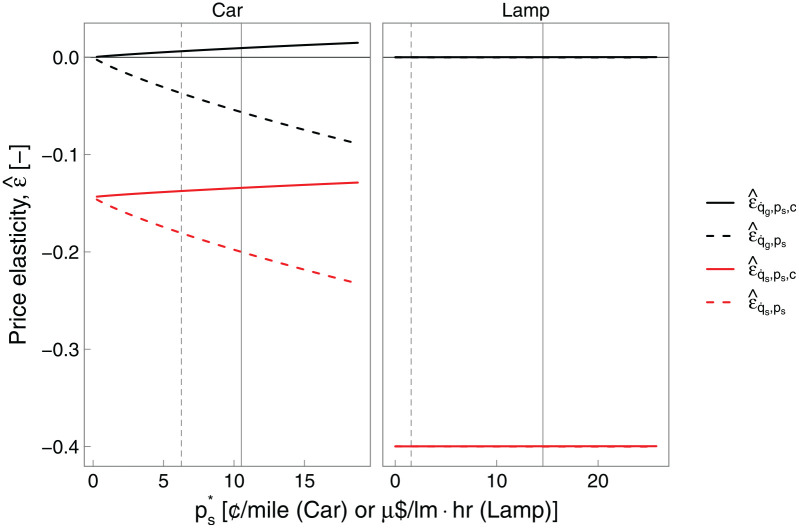
Sensitivity of post substitution effect price elasticities ( $\hat{\varepsilon }$ ) to post-EEU energy service price ( ${\tilde{p}}_{s}$ ) for the CES utility model. *Note.* This graph is a visualization of equations (200), (203), (192), and (202) of Part I. The solid vertical line indicates the original energy service price ( $p_{s}^{{}^{\circ}}$ ), and the dashed vertical line indicates the upgraded energy service price ( $p_{s}^{*}={\hat{p}}_{s}={\bar{p}}_{s}={\tilde{p}}_{s}$ ) for the two examples. See Tables 7 and 10 for $p_{s}$ in different units.

## Footnotes

1 A related, notional-only (not quantified), one-dimensional visualization of direct and indirect energy rebound (but not on expenditure or consumption planes) can be found in Figure 1 of Exadaktylos and van den Bergh (2021).

2 We exclude the case of an inferior good, whose consumption decreases as real income increases, but we note here the possibility of such behavior. This behavior would however require a different utility model besides the CES utility model, which we use throughout this analysis.

3 Maintenance cost rates for both incandescent and LED lamps are likely to be equal and negligible; lamps are usually installed and forgotten. Real-world disposal cost differences between the incandescent and LED technologies are also likely to be negligible. However, if “disposal” includes recycling processes, cost rates may be different between the two technologies due to the wide variety of materials in LED lamps compared to incandescent lamps.

4 That said, without the top-down approaches, we would have few values to compare with our total rebound ( $Re_{\mathrm{tot}}$ ).

5 Also worthy of note is that direct (microeconomic) rebound of personal transport may be the most-studied subfield in the rebound literature and likely the only topic with enough studies to enable meta-reviews such as Sorrell, Dimitropoulos and Sommerville (2009), Dimitropoulos, Oueslati and Sintek (2018), and Gillingham (2020).

6 The value of $\varepsilon_{{\overset{\cdot }{q}}_{s},p_{s}}^{{}^{\circ}}=-0.13$ was obtained via iteration: values for $\varepsilon_{{\overset{\cdot }{q}}_{s},p_{s}}^{{}^{\circ}}$ were guessed until Equation (23) of Part I gave $Re_{dsub}=4\%$ .

7 The constant price elasticity (CPE) utility model in Table 14 follows Borenstein (2015) who holds income constant, not utility, when calculating the substitution effect. Furthermore, Borenstein’s income effect assumes all post-emplacement freed cash ( ${\overset{\cdot }{N}}^{*}$ ) is spent at the energy intensity of the economy ( $I_{E}$ ).

## Appendices

### A. Nomenclature

Presentation of the rigorous analytical framework is aided by a nomenclature that describes energy stages and rebound effects. Table A.1 shows symbols and abbreviations, their meanings, and example units. Table A.2 shows Greek letters, their meanings, and example units. Table A.3 shows initialisms and acronyms. Table A.4 shows symbol decorations and their meanings. Table A.5 shows subscripts and their meanings.

**Table A.1.** Symbols and Abbreviations.

Symbol	Meaning [example units]
a	a point in the emplacement effect in rebound planes or the share parameter in the CES utility model [–]
b	a point in the emplacement effect in rebound planes
C	cost [$]
c	a point in the substitution effect in rebound planes
d	a point in the income effect on rebound planes
E	final energy [MJ]
f	expenditure share [–]
G	freed cash [$]
I	energy intensity of economic activity [MJ/$]
k	macro factor [–]
M	income [$]
N	net savings [$]
p	price [$]
Q	quantity at the macroeconomic level [–]
q	quantity [–]
Re	rebound [–]
r	real discount rate [1/yr]
S	energy cost savings [$]
t	energy conversion device lifetime [yr]
u	utility [utils]
x	the abscissa coordinate
y	the ordinate coordinate

**Table A.2.** Greek Letters.

Greek letter	Meaning [example units]
α	subscript that indicates capital cost payments at beginning of life
∆	difference (later quantity less earlier quantity, see Figure 1)
ε	price or income elasticity [–]
$\varepsilon_{{\overset{\cdot }{q}}_{s},\overset{\cdot }{M}}$	income ( $\overset{\cdot }{M}$ ) elasticity of energy service demand ( ${\overset{\cdot }{q}}_{s}$ ) [–]
$\varepsilon_{{\overset{\cdot }{q}}_{g},\overset{\cdot }{M}}$	income ( $\overset{\cdot }{M}$ ) elasticity of other goods demand ( ${\overset{\cdot }{q}}_{o}$ ) [–]
$\varepsilon_{{\overset{\cdot }{q}}_{s},p_{s}}$	uncompensated energy service price ( $p_{s}$ ) elasticity of energy service demand ( ${\overset{\cdot }{q}}_{s}$ ) [–]
$\varepsilon_{{\overset{\cdot }{q}}_{g},p_{s}}$	uncompensated energy service price ( $p_{s}$ ) elasticity of other goods demand ( ${\overset{\cdot }{q}}_{o}$ ) [–]
$\varepsilon_{{\overset{\cdot }{q}}_{s},p_{s},c}$	compensated energy service price ( $p_{s}$ ) elasticity of energy service demand ( ${\overset{\cdot }{q}}_{s}$ ) [–]
$\varepsilon_{{\overset{\cdot }{q}}_{g},p_{s},c}$	compensated energy service price ( $p_{s}$ ) elasticity of other goods demand ( ${\overset{\cdot }{q}}_{o}$ ) [–]
η	final-energy-to-service efficiency [vehicle-km/MJ]
ω	subscript that indicates disposal cost at end of life
ρ	exponent in the CES utility function, ρ≡(σ−1)/σ [–]
σ	elasticity of substitution between the energy service ( ${\overset{\cdot }{q}}_{s}^{{}^{\circ}}$ ) and other goods ( ${\overset{\cdot }{q}}_{o}^{{}^{\circ}}$ ) [–]
τ	multiplicative term that accounts for discounting [–]

**Table A.3.** Initialisms and Acronyms.

Abbreviation	Meaning
APF	aggregate production function
BOL	beginning of life
CES	constant elasticity of substitution
CGE	computable general equilibrium
CPE	constant price elasticity
CV	compensating variation
EEU	energy efficiency upgrade
EOL	end of life
EPSRC	engineering and physical sciences research council
EV	electric vehicle
GDP	gross domestic product
LAIDS	linear approximation to almost ideal demand system
LED	light emitting diode
mpg	miles per U.S. gallon
RECS	residential energy consumption survey
TVM	time value of money
UK	United Kingdom
UKRI	UK Research and Innovation
U.S.	United States
VMT	vehicle miles traveled
w.r.t.	with respect to

**Table A.4.** Decorations.

Decoration	Meaning [example units]
$X^{{}^{\circ}}$	x originally (before the emplacement effect)
$X^{*}$	x after the emplacement effect (before the substitution effect)
$\hat{X}$	x after the substitution effect (before the income effect)
$\bar{X}$	x after the income effect (before the macro effect)
$\tilde{X}$	x after the macro effect
$\overset{\cdot }{X}$	rate of x [units of X/yr]
$M^{\prime }$	effective income [$]

**Table A.5.** Subscripts.

Subscript	Meaning
0	quantity at an initial time
1	a specific point on the consumption plane
c	compensated
cap	capital costs
dev	device
dempl	direct emplacement effect
dinc	direct income effect
dir	direct effects (at the energy conversion device)
dsub	direct substitution effect
E	energy
emb	embodied
empl	emplacement effect
g	other expenditures (besides energy) by the device user
iempl	indirect emplacement effects
iinc	indirect income effect
inc	income effect
indir	indirect effects (beyond the energy conversion device)
isub	indirect substitution effect
life	lifetime
macro	macro effect
OM	operations and maintenance
OMd	operations, maintenance, and disposal
own	ownership duration
s	service stage of the energy conversion chain
sub	substitution effect
tot	sum of all rebound effects in the framework

Differences are indicated by the Greek letter Δ and always signify subtraction of a quantity at an earlier stage of Figure 1 from the same quantity at the next later stage of Figure 1. For example, $\Delta \bar{X}\;\equiv \;\bar{X}-\hat{X}$ , and $\Delta \tilde{X}\;\equiv \;\tilde{X}-\bar{X}$ . Lack of decoration on a difference term indicates a difference that spans all stages of Figure 1. For example, $\Delta X\;\equiv \;\tilde{X}-X^{{}^{\circ}}$ . ΔX is also the sum of differences across each stage in Figure 1, as shown below.

(1)
$$\begin{array}{c} \Delta X\;=\Delta \tilde{X}+\Delta \bar{X}+\Delta \hat{X}+\Delta X^{*}\\ \Delta X\;=\left(\tilde{X}-\bar{X}\right)+\left(\bar{X}-\hat{X}\right)+\left(\hat{X}-X^{*}\right)+\left(X^{*}-X^{{}^{\circ}}\right)\\ \Delta X\;=(\tilde{X}-\bar{X}/)+\left(\bar{X}/-\hat{X}/\right)+\left(\hat{X}/-X^{*}/\;\right)+\left(X^{*}/-X^{{}^{\circ}}\right)\\ \Delta X\;=\tilde{X}-X^{{}^{\circ}} \end{array}$$

### B. Mathematical Details of Rebound Planes

Rebound planes show the impact of direct and indirect rebound effects for energy, expenditure, and consumption aspects. Rebound planes for the car example can be found in Figures 2 to 4. Rebound planes for the lamp example can be found in Figures 5 to 7.

This appendix shows the mathematical details of rebound planes, specifically derivations of equations for lines and curves shown in Table B.1. The lines and curves enable construction of numerically precise and accurate paths in rebound planes as shown in Figures 2 to 7.

**Table B.1.** Lines and Curves for Rebound Planes.

Rebound plane	Lines and curves
Energy	Constant total energy consumption lines
	0% and 100% rebound lines
Expenditure	Constant expenditure lines
Consumption	Constant expenditure lines
	Rays from origin to ∧ point
	Indifference curves

#### B.1. Energy Planes

The energy plane shows direct (on the x -axis) and indirect (on the y -axis) energy consumption associated with the energy conversion device and the device user. Lines of total energy consumption isoquants provide a scale for total rebound. For example, the 0% and 100% rebound lines are constant total energy consumption lines which pass through the original point (°) and the post-direct-emplacement-effect point ( a ) in the energy plane.

The equation of a constant total energy consumption line is derived from

(2)
$${\overset{\cdot }{E}}_{\mathrm{tot}}={\overset{\cdot }{E}}_{\mathrm{dir}}+{\overset{\cdot }{E}}_{\mathrm{indir}}$$

at any rebound stage. Direct energy consumption is energy consumed by the energy conversion device ( ${\overset{\cdot }{E}}_{s}$ ), and indirect energy consumption is the sum of embodied energy, energy associated with maintenance and disposal, and energy associated with expenditures on other goods ( ${\overset{\cdot }{E}}_{\mathrm{emb}}+\left({\overset{\cdot }{C}}_{\mathrm{OMd}}+{\overset{\cdot }{C}}_{g}\right)I_{E}$ ).

For the energy plane, direct energy consumption is placed on the x -axis and indirect energy consumption is placed on the y -axis. To derive the equation of a constant energy consumption line, we first rearrange to put the y coordinate on the left of the equation:

(3)
$${\overset{\cdot }{E}}_{\mathrm{indir}}=-{\overset{\cdot }{E}}_{\mathrm{dir}}+{\overset{\cdot }{E}}_{\mathrm{tot}}\;.$$

Next, we substitute y for ${\overset{\cdot }{E}}_{\mathrm{indir}}$ , x for ${\overset{\cdot }{E}}_{\mathrm{dir}}$ , and ${\overset{\cdot }{E}}_{s}+{\overset{\cdot }{E}}_{\mathrm{emb}}+\left({\overset{\cdot }{C}}_{\mathrm{OMd}}+{\overset{\cdot }{C}}_{g}\right)I_{E}$ for ${\overset{\cdot }{E}}_{\mathrm{tot}}$ to obtain

(4)
$$y=-x+{\overset{\cdot }{E}}_{s}+{\overset{\cdot }{E}}_{\mathrm{emb}}+\left({\overset{\cdot }{C}}_{\mathrm{OMd}}+{\overset{\cdot }{C}}_{g}\right)I_{E}\;,$$

where all of ${\overset{\cdot }{E}}_{s}$ , ${\overset{\cdot }{E}}_{\mathrm{emb}}$ , ${\overset{\cdot }{C}}_{\mathrm{OMd}}$ , and ${\overset{\cdot }{C}}_{g}$ apply at the same rebound stage.

The constant total energy consumption line that passes through the original point (°) shows 100% rebound:

(5)
$$y=-x+\overset{.}{E_{s}^{{}^{\circ}}}+{\overset{\cdot }{E}}_{\mathrm{emb}}^{{}^{\circ}}+\left({\overset{\cdot }{C}}_{\mathrm{OMd}}^{{}^{\circ}}+{\overset{\cdot }{C}}_{g}^{{}^{\circ}}\right)I_{E}\;.$$

The 0% rebound line is the constant total energy consumption line that accounts for expected energy savings ( $\overset{\cdot }{S}\mathrm{dev}$ ) only:

(6)
$$y=-x+\left({\overset{\cdot }{E}}_{s}^{{}^{\circ}}-{\overset{\cdot }{S}}_{\mathrm{dev}}\right)+{\overset{\cdot }{E}}_{\mathrm{emb}}^{{}^{\circ}}+\left({\overset{\cdot }{C}}_{\mathrm{OMd}}^{{}^{\circ}}+{\overset{\cdot }{C}}_{g}^{{}^{\circ}}\right)I_{E}\;.$$

The above line passes through the a point in the energy plane.

#### B.2. Expenditure Planes

The expenditure plane shows direct (on the x -axis) and indirect (on the y -axis) expenses associated with the energy conversion device and the device user. Lines of constant expenditure are important, because they provide budget constraints for the device user.

The equation of a constant total expenditure line is derived from the budget constraint

(7)
$${\overset{\cdot }{C}}_{\mathrm{tot}}={\overset{\cdot }{C}}_{\mathrm{dir}}+{\overset{\cdot }{C}}_{\mathrm{indir}}$$

at any rebound stage. In the expenditure plane, indirect expenditures are placed on the y -axis and direct expenditures on energy for the energy conversion device are place on the x -axis. Direct expenditure is the cost of energy consumed by the energy conversion device ( ${\overset{\cdot }{C}}_{s}=p_{E}{\overset{\cdot }{E}}_{s}$ ), and indirect expenses are the sum of capital costs, operations, maintenanace, and disposal costs, and expenditures on other goods ( $\tau_{\alpha }{\overset{\cdot }{C}}_{\mathrm{cap}}+{\overset{\cdot }{C}}_{\mathrm{OMd}}+{\overset{\cdot }{C}}_{g}$ ). Rearranging to put the y -axis variable on the left side of the equation gives

(8)
$${\overset{\cdot }{C}}_{\mathrm{indir}}=-{\overset{\cdot }{C}}_{\mathrm{dir}}+{\overset{\cdot }{C}}_{\mathrm{tot}}\;.$$

Substituting y for ${\overset{\cdot }{C}}_{\mathrm{indir}}$ , x for ${\overset{\cdot }{C}}_{\mathrm{dir}}$ , and ${\overset{\cdot }{C}}_{s}+\tau_{\alpha }{\overset{\cdot }{C}}_{\mathrm{cap}}+{\overset{\cdot }{C}}_{\mathrm{OMd}}+{\overset{\cdot }{C}}_{g}$ for ${\overset{\cdot }{C}}_{\mathrm{tot}}$ gives

(9)
$$y=-x+{\overset{\cdot }{C}}_{s}+\tau_{\alpha }{\overset{\cdot }{C}}_{\mathrm{cap}}+{\overset{\cdot }{C}}_{\mathrm{OMd}}+{\overset{\cdot }{C}}_{g}\;,$$

where all of ${\overset{\cdot }{C}}_{s}$ , $\tau_{\alpha }$ , ${\overset{\cdot }{C}}_{\mathrm{cap}}$ , ${\overset{\cdot }{C}}_{\mathrm{OMd}}$ , and ${\overset{\cdot }{C}}_{g}$ apply at the same rebound stage.

The constant total expenditure line that passes through the original point (°) shows the budget constraint for the device user:

(10)
$$y=-x+\overset{.}{C_{s}^{{}^{\circ}}}+\tau_{\alpha }^{{}^{\circ}}{\overset{\cdot }{C}}_{\mathrm{cap}}^{{}^{\circ}}+\overset{.}{C_{\mathrm{OMd}}^{{}^{\circ}}}+\overset{.}{C_{g}^{{}^{\circ}}}\;,$$

into which equation (78) of Part I can be substituted with ${\overset{\cdot }{C}}_{s}^{{}^{\circ}}=p_{E}\overset{\cdot }{E}{{}^{\circ}}_{s}$ and $\overset{\cdot }{N}{}^{\circ}=0$ to obtain

(11)
$$y=-x+\overset{\cdot }{M}\;.$$

The constant total expenditure line that accounts for expected energy savings ( $\overset{\cdot }{S}\mathrm{dev}$ ) and freed cash ( $\overset{\cdot }{G}=p_{E}{\overset{\cdot }{S}}_{\mathrm{dev}}$ ) only is given by:

(12)
$$y=-x+\left({\overset{\cdot }{C}}_{s}^{{}^{\circ}}-\overset{\cdot }{G}\right)+\tau_{\alpha }^{{}^{\circ}}{\overset{\cdot }{C}}_{\mathrm{cap}}^{{}^{\circ}}+{\overset{\cdot }{C}}_{\mathrm{OMd}}^{{}^{\circ}}+\overset{.}{C_{g}^{{}^{\circ}}}\;,$$

or

(13)
$$y=-x+\overset{\cdot }{M}-\overset{\cdot }{G}\;.$$

The line given by the equation above passes through the a point in the expenditure plane.

#### B.3. Consumption Planes

The consumption plane shows expenditures in the ${\overset{\cdot }{C}}_{g}/{\overset{\cdot }{C}}_{g}^{{}^{\circ}}$ versus ${\overset{\cdot }{q}}_{s}/{\overset{\cdot }{q}}_{s}^{{}^{\circ}}$ plane, according to the utility model. (See Appendix C of Part I.) Consumption planes include (i) constant expenditure lines given prices, (ii) a ray from the origin through the ∧ point, and (iii) indifference curves. Derivations for each are shown in the following subsections.

##### B.3.1. Constant expenditure lines

There are four constant expenditure lines in the consumption planes of Figures 4 and 7. The constant expenditure lines pass through the original point (line °—°), the post-emplacement point (line *—*), the post-substitution point (line ∧—∧), and the post-income point (line −—−). Similar to the expenditure plane, lines of constant expenditure in the consumption plane are derived from the budget constraint of the device user at each of the four points.

Prior to the EEU, the budget constraint is given by equation (78) of Part I. Substituting $p_{s}^{{}^{\circ}}{\overset{\cdot }{q}}_{s}^{{}^{\circ}}$ for $p_{E}\overset{.}{E_{s}^{{}^{\circ}}}$ and recognizing that there is no net savings before the EEU ( ${\overset{\cdot }{N}}^{{}^{\circ}}=0$ ) gives

(14)
$$\overset{\cdot }{M}=p_{s}^{{}^{\circ}}{\overset{\cdot }{q}}_{s}^{{}^{\circ}}+\tau_{\alpha }^{{}^{\circ}}{\overset{\cdot }{C}}_{\mathrm{cap}}^{{}^{\circ}}+\overset{.}{C_{\mathrm{OMd}}^{{}^{\circ}}}+{\overset{\cdot }{C}}_{g}^{{}^{\circ}}\;.$$

To create the line of constant expenditure in the consumption plane, we allow ${\overset{\cdot }{q}}_{s}^{{}^{\circ}}$ and ${\overset{\cdot }{C}}_{g}^{{}^{\circ}}$ to vary in a compensatory manner: when one increases, the other must decrease. To show that variation along the constant expenditure line, we remove the notation that ties ${\overset{\cdot }{q}}_{s}^{{}^{\circ}}$ and $\overset{.}{C_{g}^{{}^{\circ}}}$ to the original point (°) to obtain

(15)
$$\overset{\cdot }{M}=p_{s}^{{}^{\circ}}{\overset{\cdot }{q}}_{s}+\tau_{\alpha }^{{}^{\circ}}\overset{.}{C_{\mathrm{cap}}^{{}^{\circ}}}+{\overset{\cdot }{C}}_{\mathrm{OMd}}^{{}^{\circ}}+{\overset{\cdot }{C}}_{g}\;,$$

where all of $\overset{\cdot }{M}$ , $p_{s}^{{}^{\circ}}$ , $\tau_{\alpha }^{{}^{\circ}}{\overset{\cdot }{C}}_{\mathrm{cap}}^{{}^{\circ}}$ , and ${\overset{\cdot }{C}}_{\mathrm{OMd}}^{{}^{\circ}}$ apply at the same rebound stage, namely the original point (°) in this instance.

To derive the equation of the line representing the original budget constraint in ${\overset{\cdot }{C}}_{g}/\overset{.}{C_{g}^{{}^{\circ}}}$ versus ${\overset{\cdot }{q}}_{s}/{\overset{\cdot }{q}}_{s}^{{}^{\circ}}$ space (the °—° line through the ° point in consumption planes), we solve for ${\overset{\cdot }{C}}_{g}$ to obtain

(16)
$${\overset{\cdot }{C}}_{g}=-p_{s}^{{}^{\circ}}{\overset{\cdot }{q}}_{s}+\overset{\cdot }{M}-\tau_{\alpha }^{{}^{\circ}}{\overset{\cdot }{C}}_{\mathrm{cap}}^{{}^{\circ}}-{\overset{\cdot }{C}}_{\mathrm{OMd}}^{{}^{\circ}}\;.$$

Multiplying judiciously by ${\overset{\cdot }{C}}_{g}^{{}^{\circ}}/\overset{.}{C_{g}^{{}^{\circ}}}$ and ${\overset{\cdot }{q}}_{s}^{{}^{\circ}}/{\overset{\cdot }{q}}_{s}^{{}^{\circ}}$ gives

(17)
$$\frac{{\overset{\cdot }{C}}_{g}}{\overset{.}{C_{g}^{{}^{\circ}}}}\overset{.}{C_{g}^{{}^{\circ}}}=-p_{s}^{{}^{\circ}}\frac{{\overset{\cdot }{q}}_{s}}{{\overset{\cdot }{q}}_{s}^{{}^{\circ}}}{\overset{\cdot }{q}}_{s}^{{}^{\circ}}+\overset{\cdot }{M}-\tau_{\alpha }^{{}^{\circ}}{\overset{\cdot }{C}}_{\mathrm{cap}}^{{}^{\circ}}-{\overset{\cdot }{C}}_{\mathrm{OMd}}^{{}^{\circ}}\;.$$

Dividing both sides by $\overset{\cdot }{C}{{}^{\circ}}_{g}$ yields

(18)
$$\frac{{\overset{\cdot }{C}}_{g}}{{\overset{\cdot }{C}}_{g}^{{}^{\circ}}}=-\frac{p_{s}^{{}^{\circ}}{\overset{\cdot }{q}}_{s}^{{}^{\circ}}}{{\overset{\cdot }{C}}_{g}^{{}^{\circ}}}\frac{{\overset{\cdot }{q}}_{s}}{{\overset{\cdot }{q}}_{s}^{{}^{\circ}}}+\frac{1}{\overset{.}{C_{g}^{{}^{\circ}}}}\left(\overset{\cdot }{M}-\tau_{\alpha }^{{}^{\circ}}{\overset{\cdot }{C}}_{\mathrm{cap}}^{{}^{\circ}}-{\overset{\cdot }{C}}_{\mathrm{OMd}}^{{}^{\circ}}\right)\;.$$

Noting that ${\overset{\cdot }{q}}_{s}/{\overset{\cdot }{q}}_{s}^{{}^{\circ}}$ and ${\overset{\cdot }{C}}_{g}/{\overset{\cdot }{C}}_{g}^{{}^{\circ}}$ are the x -axis and y -axis, respectively, of the consumption plane gives

(19)
$$y=-\frac{p_{s}^{{}^{\circ}}\overset{.}{q_{s}^{{}^{\circ}}}}{{\overset{\cdot }{C}}_{g}^{{}^{\circ}}}x+\frac{1}{\overset{.}{C_{g}^{{}^{\circ}}}}\left(\overset{\cdot }{M}-\tau_{\alpha }^{{}^{\circ}}\overset{.}{C_{\mathrm{cap}}^{{}^{\circ}}}-{\overset{\cdot }{C}}_{\mathrm{OMd}}^{{}^{\circ}}\right)\;.$$

A similar procedure can be employed to derive the equation of the *—* line through the * point after the emplacement effect. The starting point is the budget constraint at the * point (equation (80) of Part I) with $p_{s}^{*}{\overset{\cdot }{q}}_{s}$ replacing $p_{E}\overset{.}{E_{s}^{*}}$ and ${\overset{\cdot }{C}}_{g}$ replacing ${\overset{\cdot }{C}}_{g}^{*}$ .

(20)
$$\;\overset{\cdot }{M}=p_{s}^{*}{\overset{\cdot }{q}}_{s}+\tau_{\alpha }^{*}{\overset{\cdot }{C}}_{\mathrm{cap}}^{*}+{\overset{\cdot }{C}}_{\mathrm{OMd}}^{*}+{\overset{\cdot }{C}}_{g}+\overset{\cdot }{N}*$$

Substituting equation (89) of Part I for ${\overset{\cdot }{N}}^{*}$ , substituting equation (90) of Part I to obtain $\overset{\cdot }{G}$ , multiplying judiciously by ${\overset{\cdot }{C}}_{g}^{{}^{\circ}}/{\overset{\cdot }{C}}_{g}^{{}^{\circ}}$ and ${\overset{\cdot }{q}}_{s}^{{}^{\circ}}/{\overset{\cdot }{q}}_{s}^{{}^{\circ}}$ , rearranging, and noting that ${\overset{\cdot }{q}}_{s}/{\overset{\cdot }{q}}_{s}^{{}^{\circ}}$ is the x -axis and ${\overset{\cdot }{C}}_{g}/{\overset{\cdot }{C}}_{g}^{{}^{\circ}}$ is the y -axis gives

(21)
$$y=-\frac{p_{s}^{*}{\overset{\cdot }{q}}_{s}^{{}^{\circ}}}{\overset{.}{C_{g}^{{}^{\circ}}}}x+\frac{1}{\overset{.}{C_{g}^{{}^{\circ}}}}\left(\overset{\cdot }{M}-\tau_{\alpha }^{{}^{\circ}}\overset{.}{C_{\mathrm{cap}}^{{}^{\circ}}}-{\overset{\cdot }{C}}_{\mathrm{OMd}}^{{}^{\circ}}-\overset{\cdot }{G}\right)\;.$$

Note that the slope of equation (21) is less negative than the slope of equation (19), because $p_{s}^{*}<p_{s}^{{}^{\circ}}$ . The y -intercept of equation (21) is less than the y -intercept of equation (19), reflecting freed cash. Both effects are seen in the consumption planes (Figures 4 and 7). The °—° and *—* lines intersect at the coincident ° and * points.

A similar derivation process can be used to find the equation of the line representing the budget constraint after the substitution effect (the ∧—∧ line through the ∧ point). The starting point is equation (93) of Part I, and the equation for the constant expenditure line is

(22)
$$y=-\frac{p_{s}^{*}{\overset{\cdot }{q}}_{s}^{{}^{\circ}}}{{\overset{\cdot }{C}}_{g}^{{}^{\circ}}}x+\frac{1}{{\overset{\cdot }{C}}_{g}^{{}^{\circ}}}\left(\overset{\cdot }{M}-\tau_{\alpha }^{{}^{\circ}}{\overset{\cdot }{c}}_{\mathrm{cap}}^{{}^{\circ}}-{\overset{\cdot }{C}}_{\mathrm{OMd}}^{{}^{\circ}}-\overset{\cdot }{C}+p_{s}^{*}\Delta {\hat{\overset{\cdot }{q}}}_{s}+\Delta {\hat{\overset{\cdot }{C}}}_{g}\right)\;.$$

Note that the ∧–∧ line (equation (22)) has the same slope as the *_ * line (equation (21)) but a lower y -intercept.

Finally, the corresponding derivation for the equation of the constant expenditure line through the − point (line −—−) starts with equation (102) of Part I and comes to

(23)
$$y=-\frac{\overset{.}{p_{s}^{*}}{\overset{\cdot }{q}}_{s}^{{}^{\circ}}}{\overset{.}{C_{g}^{{}^{\circ}}}}x+\frac{1}{{\overset{\cdot }{C}}_{g}^{{}^{\circ}}}\left(\overset{\cdot }{M}-\tau_{\alpha }^{{}^{\circ}}{\overset{\cdot }{C}}_{\mathrm{cap}}^{{}^{\circ}}-{\overset{\cdot }{C}}_{\mathrm{OMd}}^{{}^{\circ}}-\Delta \left(\tau_{\alpha }{\overset{\cdot }{C}}_{\mathrm{cap}}\right)*-\Delta {\overset{\cdot }{C}}_{\mathrm{OMd}}^{*}\right)\;.$$

Simplification of Δ terms gives

(24)
$$y=-\frac{{\tilde{p}}_{s}{\overset{\cdot }{q}}_{s}^{{}^{\circ}}}{{\overset{\cdot }{C}}_{g}^{{}^{\circ}}}x+\frac{1}{{\overset{\cdot }{C}}_{g}^{{}^{\circ}}}\left(\overset{\cdot }{M}-\tau_{\alpha }^{*}{\overset{\cdot }{C}}_{\mathrm{cap}}^{*}-{\overset{\cdot }{C}}_{\mathrm{OMd}}^{*}\right)\;.$$

##### B.3.2. Ray from the origin to the ∧ point

In the consumption plane, the ray from the origin to the ∧ point (line r—r) defines the path along which the income effect (lines ^  *d* and *d*  –) operates. The ray from the origin to the ∧ point has slope $\left({\hat{\overset{\cdot }{C}}}_{g}/{\overset{\cdot }{C}}_{g}^{{}^{\circ}}\right)/\left({\hat{\overset{\cdot }{q}}}_{s}/{\overset{\cdot }{q}}_{s}^{{}^{\circ}}\right)$ and a y -intercept of 0. Therefore, the equation of line r—r is

(25)
$$y=\frac{{\hat{\overset{\cdot }{C}}}_{g}/{\overset{\cdot }{C}}_{g}^{{}^{\circ}}}{{\hat{\overset{\cdot }{q}}}_{s}/{\overset{\cdot }{q}}_{s}^{{}^{\circ}}}\;x\;.$$

##### B.3.3. Indifference curves

In the consumption plane, indifference curves represent lines of constant utility for the energy conversion device user. In the consumption plane ( ${\overset{\cdot }{C}}_{g}/{\overset{\cdot }{C}}_{g}^{{}^{\circ}}$ vs. ${\overset{\cdot }{q}}_{s}/{\overset{\cdot }{q}}_{s}^{{}^{\circ}}$ ), any indifference curve is given by equation (159) of Part I with $f_{\overset{\cdot }{C}s}^{{}^{\circ}}$ replacing the share parameter a , as shown in Appendix C of Part I. Recognizing that ${\overset{\cdot }{C}}_{g}/{\overset{\cdot }{C}}_{g}^{{}^{\circ}}$ is on the y -axis and ${\overset{\cdot }{q}}_{s}/{\overset{\cdot }{q}}_{s}^{{}^{\circ}}$ is on the x -axis leads to substitution of y for ${\overset{\cdot }{C}}_{g}/{\overset{\cdot }{C}}_{g}^{{}^{\circ}}$ and x for ${\overset{\cdot }{q}}_{s}/{\overset{\cdot }{q}}_{s}^{{}^{\circ}}$ to obtain

(26)
$$y={\left[\frac{1}{1-f_{{\overset{\cdot }{C}}_{s}}^{{}^{\circ}}}{\left(\frac{\overset{\cdot }{u}}{{\overset{\cdot }{u}}^{{}^{\circ}}}\right)}^{\rho }-\frac{f_{{\overset{\cdot }{C}}_{s}}^{{}^{\circ}}}{1-f_{{\overset{\cdot }{C}}_{s}}^{{}^{\circ}}}{\left(x\right)}^{\rho }\right]}^{\left(1/\rho \right)}\;.$$

At any point on the ${\overset{\cdot }{C}}_{g}/\overset{.}{C_{g}^{{}^{\circ}}}$ versus ${\overset{\cdot }{q}}_{s}/{\overset{\cdot }{q}}_{s}^{{}^{\circ}}$ plane, namely ( ${\overset{\cdot }{q}}_{s,1}/{\overset{\cdot }{q}}_{s}^{{}^{\circ}}$ , ${\overset{\cdot }{C}}_{g,1}/{\overset{\cdot }{C}}_{g}^{{}^{\circ}}$ ), indexed utility ( ${\overset{\cdot }{u}}_{1}/{\overset{\cdot }{u}}^{{}^{\circ}}$ ) is given by equation (16) of Part I as

(27)
$$\frac{{\overset{\cdot }{u}}_{1}}{{\overset{\cdot }{u}}^{{}^{\circ}}}={\left[f_{{\overset{\cdot }{C}}_{s}}^{{}^{\circ}}{\left(\frac{{\overset{\cdot }{q}}_{s,1}}{{\overset{\cdot }{q}}_{s}^{{}^{\circ}}}\right)}^{\rho }+\left(1-f_{{\overset{\cdot }{C}}_{s}}^{{}^{\circ}}\right){\left(\frac{{\overset{\cdot }{C}}_{g,1}}{{\overset{\cdot }{C}}_{g}^{{}^{\circ}}}\right)}^{\rho }\right]}^{\left(1/\rho \right)}\;.$$

Substituting equation (27) into equation (26) for $\overset{\cdot }{u}/{\overset{\cdot }{u}}^{{}^{\circ}}$ and simplifying exponents gives

(28)
$$y={\left\{\frac{1}{1-f_{{\overset{\cdot }{C}}_{s}}^{{}^{\circ}}}\left[f_{{\overset{\cdot }{C}}_{s}}^{{}^{\circ}}{\left(\frac{{\overset{\cdot }{q}}_{s,1}}{{\overset{\cdot }{q}}_{s}^{{}^{\circ}}}\right)}^{\rho }+\left(1-f_{{\overset{\cdot }{C}}_{s}}^{{}^{\circ}}\right){\left(\frac{{\overset{\cdot }{C}}_{g,1}}{{\overset{\cdot }{C}}_{g}^{{}^{\circ}}}\right)}^{\rho }\right]-\frac{{\overset{\cdot }{f}}_{{\overset{\cdot }{C}}_{s}}^{{}^{\circ}}}{1-f_{{\overset{\cdot }{C}}_{s}}^{{}^{\circ}}}{\left(x\right)}^{\rho }\right\}}^{\left(1/\rho \right)}.$$

Simplifying further yields the equation of an indifference curve passing through point ( ${\overset{\cdot }{q}}_{s,1}/{\overset{\cdot }{q}}_{s}^{{}^{\circ}}$ , ${\overset{\cdot }{C}}_{g,1}/{\overset{\cdot }{C}}_{g}^{{}^{\circ}}$ ):

(29)
$$y={\left\{\left(\frac{f_{{\overset{\cdot }{C}}_{s}}^{{}^{\circ}}}{1-f_{{\overset{\cdot }{C}}_{s}}^{{}^{\circ}}}\right)\left[{\left(\frac{{\overset{\cdot }{q}}_{s,1}}{{\overset{\cdot }{q}}_{s}^{{}^{\circ}}}\right)}^{\rho }-{\left(x\right)}^{\rho }\right]+{\left(\frac{{\overset{\cdot }{C}}_{g,1}}{{\overset{\cdot }{C}}_{g}^{{}^{\circ}}}\right)}^{\rho }\right\}}^{\left(1/\rho \right)}\;.$$

Note that if x is ${\overset{\cdot }{q}}_{s,1}/{\overset{\cdot }{q}}_{s}^{{}^{\circ}}$ , y becomes ${\overset{\cdot }{C}}_{g,1}/{\overset{\cdot }{C}}_{g}^{{}^{\circ}}$ , as expected.

### C. Univariate Sensitivity Analyses

Sensitivity analyses show the effect of independently varied parameters on total rebound and rebound components. In the context of this framework, sensitivity analyses can show important trends, tendencies, and relationships between rebound parameters and rebound magnitudes. Key rebound parameters include post-EEU efficiency ( $\eta^{*}$ ), post-EEU capital cost ( $C_{\mathrm{cap}}^{*}$ ), energy price ( $p_{E}$ ), pre-EEU uncompensated price elasticity of energy service demand ( $\varepsilon_{{\overset{\cdot }{q}}_{s},p_{s}}^{{}^{\circ}}$ ), the macro factor ( k ), and post-EEU energy service price ( $p_{s}^{*}$ ). Univariate sensitivity analyses (the kind shown here) should be interpreted carefully, because some rebound parameters are not expected to be independent from others.

#### C.1. Effect of Post-EEU Efficiency ( $\eta^{*}$ ) on Rebound Terms

Figure C.1 shows that both the energy takeback rate and expected energy savings ( ${\overset{\cdot }{S}}_{\mathrm{dev}}$ ) increase with post-EEU efficiency ( $\eta^{*}$ ), but the relationship is asymptotic. Each unit increase of fuel economy or lighting efficiency is less effective than the previous unit increase of fuel economy or lighting efficiency for saving energy. At very high levels of fuel economy or lighting efficiency, a unit increase leads to almost no additional energy savings. Thus, we can say there are diminishing returns of fuel economy and lighting efficiency, leading to saturation of energy savings at very high levels of fuel economy and lighting efficiency. A simple example illustrates. A $\eta^{{}^{\circ}}=25$ mpg car drives $q_{s}^{{}^{\circ}}=100$ miles using $E_{s}^{{}^{\circ}}=4$ gallons of gasoline. A more-efficient car ( $\eta^{*}=30$ mpg) is expected to use $E_{s}^{*}=3.33$ gallons to drive the same distance, a savings of ${\overset{\cdot }{S}}_{\mathrm{dev}}=0.67$ gallons. Another 5 mpg boost in efficiency (to $\eta^{*}=35$ mpg) will use $E_{s}^{*}=2.86$ gal to drive 100 miles, a further expected savings of only ${\overset{\cdot }{S}}_{\mathrm{dev}}=0.47$ gallons. Each successive 5 mpg boost in fuel economy saves less energy than the previous 5 mpg boost in fuel economy.

Saturation can be seen mathematically, too. Taking the limit as $\eta^{*}\to \infty$ in equation (12) of Part I gives ${\overset{\cdot }{S}}_{\mathrm{dev}}={\overset{\cdot }{E}}_{s}^{{}^{\circ}}$ , not ∞ . Thus, efficiency saturation must occur. Figure C.1 shows that this framework correctly replicates expected efficiency saturation trends.

Saturation is especially noticeable in the lamp example compared to the car example, the difference being that the LED lamp is already much more efficient than the incandescent lamp (9.26×), whereas the hybrid car is only 1.68×more efficient than the conventional gasoline car. Thus, at $\eta^{*}=81.8$ lmċhr/Wċhr, the energy efficient LED is far closer to efficiency saturation than the hybrid vehicle (at $\eta^{*}=42$ mpg). As a result, further increases in the LED lamp’s efficiency are less effective than further increases in the hybrid car’s efficiency.

That said, actual savings is the difference between the expected energy savings line (solid line) and the takeback line (dashed line) in Figure C.1. Because the gap between the lines grows, higher efficiency yields greater energy savings, even after accounting for rebound effects. But the actual savings are always less than expected savings, due to takeback.

Figure C.1 shows that expected energy savings ( ${\overset{\cdot }{S}}_{\mathrm{dev}}$ ) increase faster than takeback as $\eta^{*}$ increases. Thus, total rebound ( $Re_{\mathrm{tot}}$ , the ratio of takeback rate to expected energy savings rate in equation (3) of Part I), decreases as efficiency grows. The lamp exhibits a relatively smaller rebound decline with efficiency, because the lamp example is closer to saturation than the car example.

Figure C.2 shows the variation of all rebound components with post-EEU efficiency ( $\eta^{*}$ ). In the car and lamp examples, direct substitution rebound ( $Re_{\mathrm{dsub}}$ ) is the rebound component most sensitive to changes in post-EEU efficiency ( $\eta^{*}$ ).

Note that the sensitivity analysis on post-upgrade efficiency ( $\eta^{*}$ , Figure C.2) is the only sensitivity analysis that requires careful explication of both the numerator and denominator of equation (3) in Part I, as in Figure C.1, because both the numerator and denominator of equation (3) in Part I change when post-upgrade efficiency ( $\eta^{*}$ ) changes. The denominator of equation (3) in Part I doesn’t change for the sensitivity analyses of Figures C.3 to C.6. Thus, for the remaining sensitivity analyses, when the rebound percentage increases (decreases), the energy takeback rate in the numerator of equation (3) in Part I increases (decreases) proportionally, and the actual energy savings rate decreases (increases) accordingly.

#### C.2. Effect of Capital Cost ( $C_{\mathrm{cap}}^{\;*}$ ) on Rebound Terms

The sensitivity of energy rebound to capital cost ( $C_{\mathrm{cap}}^{*}$ ) is shown in Figure C.3. All other things being equal, as capital cost of the EEU rises, less net savings result from the emplacement effect, leading to smaller income, macro, and total rebound. The same effects would be observed with increasing operations and maintenance ( ${\overset{\cdot }{C}}_{\mathrm{OM}}^{*}$ ) and disposal cost ( ${\overset{\cdot }{C}}_{d}^{*}$ ) rates.

#### C.3. Effect of Energy Price ( $p_{E}$ ) on Rebound Terms

The effect of energy price on rebound is shown in Figure C.4. Increasing energy prices lead to larger total rebound ( $Re_{\mathrm{tot}}$ ), because higher energy prices lead to more net savings ( $\hat{\overset{\cdot }{N}}$ ) to be spent by the device user. All other things being equal, more net savings leads to more spending on other goods and services that demand energy.

Figure C.4 also shows the effect of energy price ( $p_{E}$ ) on all rebound components. Most rebound components increase with energy price, with the car and lamp examples exhibiting different sensitivities. Substitution effects ( $Re_{\mathrm{dsub}}$ and $Re_{\mathrm{isub}}$ ) are the only rebound components that decrease with energy price ( $p_{E}$ ). Substitution effects decrease with energy price, because at high energy price, less behavior adjustment is needed to re-equilibrate after emplacement of the efficient device.

In Figure C.4, German energy prices^8^ are shown as vertical lines, providing an indication of possible energy price variations. All other things being equal, if U.S. residents paid Germany’s energy prices, total energy rebound ( $Re_{\mathrm{tot}}$ ) would be 93.0% for the car example and 148.0% for the lamp example.

#### C.4. Effect of Original Uncompensated Own Price Elasticity ( $\left(\varepsilon_{{\dot{q}}_{s},p_{s}}^{ο}\right)$ ) on Rebound Terms

Figure C.5 shows the variation of total rebound ( $Re_{\mathrm{tot}}$ ) with the original uncompensated price elasticity of energy service demand ( $\varepsilon_{{\overset{\cdot }{q}}_{s},p_{s}}^{{}^{\circ}}$ ). The effect is exponential, and total rebound increases with larger negative values of $\varepsilon_{{\overset{\cdot }{q}}_{s},p_{s}}^{{}^{\circ}}$ , as expected. The lamp example also shows stronger exponential variation than the car example. The main reason that total rebound values are different between the two examples is the larger absolute value of original uncompensated own price elasticity ( $\varepsilon_{{\overset{\cdot }{q}}_{s},p_{s}}^{{}^{\circ}}$ ) for the lamp ( −0.4 ) compared to the car ( −0.2 ). Were the car to have the same original uncompensated own price elasticity as the lamp (i.e., −0.4 ), total rebound would be closer for both examples (73.4% for the car and 67.0% for the lamp). Figure C.5 shows that direct substitution rebound ( $Re_{\mathrm{dsub}}$ ) is the most sensitive rebound component to changes in $\varepsilon_{{\overset{\cdot }{q}}_{s},p_{s}}^{{}^{\circ}}$ . For the lamp example, indirect income rebound ( $Re_{\mathrm{iinc}}$ ) also increases substantially with $\varepsilon_{{\overset{\cdot }{q}}_{s},p_{s}}^{{}^{\circ}}$ , because net savings increases substantially with $\varepsilon_{{\overset{\cdot }{q}}_{s},p_{s}}^{{}^{\circ}}$ .

#### C.5. Effect of Macro Factor ( k ) on Rebound Terms

The sensitivity of energy rebound to the macro factor ( k ) is shown in Figure C.6. The macro factor has a linear effect on total rebound ( $Re_{\mathrm{tot}}$ ) through the macro rebound component ( $Re_{\mathrm{macro}}$ ). All other rebound components are constant when k is varied independently.

#### C.6. Effect of Discount Rate ( r ) on Rebound Terms

The effect of discount rate on rebound is shown in Figure C.7. Discounting has little effect on rebound terms compared to other parameters such as upgraded efficiency ( $\eta^{*}$ , Figure C.2), capital cost ( $C_{\mathrm{cap}}^{*}$ , Figure C.3), energy price ( $p_{E}$ , Figure C.4), and own price elasticity of energy service demand ( $\varepsilon_{{\overset{\cdot }{q}}_{s},p_{s}}^{{}^{\circ}}$ , Figure C.5).

#### C.7. Effect of Energy Service Price ( $p_{s}^{*}$ ) on Price Elasticities ( $\hat{\varepsilon }$ )

The sensitivity of post-substitution effect price elasticities ( $\hat{\varepsilon }$ ) to post-upgrade energy service price ( ${\tilde{p}}_{s}$ ) is shown in Figure C.8 for the CES utility model described in Section 2.5.2 and Appendix C of Part I. Note that the left side of each graph ( $p_{s}^{*}=0$ ) represents unattainable infinite efficiency ( $\eta_{s}^{*}\to \infty$ ), that is, delivery of the energy service without energy consumption.

First, note the sign of the elasticities. As expected, both of the uncompensated price elasticities ( ${\hat{\varepsilon }}_{{\overset{\cdot }{q}}_{s},p_{s}}$ and ${\hat{\varepsilon }}_{{\overset{\cdot }{q}}_{g},\mathrm{ps}}$ , dashed lines in Figure C.8) are negative, regardless of the energy service price ( $p_{s}^{*}$ ): a lower price means more consumption of both goods, all other things being equal. The compensated own price elasticity ( ${\hat{\varepsilon }}_{{\overset{\cdot }{q}}_{s},p_{s},c}$ ) is negative and the compensated cross price elasticity ( ${\hat{\varepsilon }}_{{\overset{\cdot }{q}}_{g},p_{s},c}$ ) is positive. As $p_{s}^{*}$ declines, the consumers substitutes the energy service for other goods.

Second, the magnitude of price elasticities varies. Figure C.8 shows that the car example exhibits more variation of price elasticities ( $\hat{\varepsilon }$ ) with energy service price ( $p_{s}^{*}$ ) than the lamp example, because the expenditure share ( $f_{{\overset{\cdot }{C}}_{s}}^{{}^{\circ}}$ ) for the lamp example is very small compared to the car example. Using the constant price elasticity (CPE) utility model may be a good enough approximation in the lamp example. However, for the car example, using the CES utility function will be necessary to eliminate errors that will be present in the CPE approximation. This result is an important finding that should encourage analysts implementing analytical rebound calculations with substitution and income effects to prefer the CES utility model over the CPE approximation.

Figure C.8 shows that as efficiency increases (and $p_{s}^{*}$ decreases), the absolute value of the uncompensated price elasticities ( ${\hat{\varepsilon }}_{{\overset{\cdot }{q}}_{s},p_{s}}$ and ${\hat{\varepsilon }}_{{\overset{\cdot }{q}}_{g},p_{s}}$ ) decreases, a change that exceeds the slightly increasing (in absolute value terms) compensated own price elasticity ( ${\hat{\varepsilon }}_{{\overset{\cdot }{q}}_{s},p_{s},c}$ ). Thus, direct rebound is attenuated as efficiency increases, relative to a constant price elasticity model. (See also the patterns of lines of Figure C.2, which show a declining trend.)
